# Characterization of proliferation, differentiation potential, and gene expression among clonal cultures of human dental pulp cells

**DOI:** 10.1007/s13577-020-00327-9

**Published:** 2020-03-16

**Authors:** Tomoko Kobayashi, Daisuke Torii, Takanori Iwata, Yuichi Izumi, Masanori Nasu, Takeo W. Tsutsui

**Affiliations:** 1 Research Center for Odontology, School of Life Dentistry at Tokyo, The Nippon Dental University, 1-9-20 Fujimi, Chiyoda-ku, Tokyo, 102-8159 Japan; 2 Department of Periodontology, Graduate School of Medical and Dental Sciences, Tokyo Medical and Dental University (TMDU), 1-5-45 Yushima, Bunkyo-ku, Tokyo, 113-8510 Japan; 3 Department of Pharmacology, School of Life Dentistry at Tokyo, The Nippon Dental University, 1-9-20 Fujimi, Chiyoda-ku, Tokyo, 102-8159 Japan; 4 Oral Care Perio Center, Southern TOHOKU General Hospital, Southern TOHOKU Research Institute for Neuroscience, 7-115, Yatsuyamada, Koriyama City, Fukushima 963-8563 Japan

**Keywords:** Mesenchymal stem cells (MSCs), Dental pulp, Differentiation, Gene expression profiles, Surface marker genes

## Abstract

Mesenchymal stem cells are a highly promising source of cells for regeneration therapy because of their multilineage differentiation potential. However, distinct markers for mesenchymal stem cells are not well-established. To identify new candidate marker genes for multipotent human dental pulp stem cells, we analyzed the characteristics and gene expression profiles of cell clones obtained from a single dental pulp specimen derived from an 11-year-old female patient. Fifty colony-forming single cell-derived clones were separately cultured until the cessation of growth. These clones varied in their proliferation abilities and surface marker (STRO-1 and CD146) expression patterns, as well as their odontogenic, adipogenic, and chondrogenic differentiation potentials. Four clones maintained their original differentiation potentials during long-term culture. Gene expression profile by DNA microarray analysis of five representative clones identified 1227 genes that were related to multipotency. Ninety of these 1227 genes overlapped with genes reportedly involved in ‘stemness or differentiation’. Based on the predicted locations of expressed protein products and large changes in expression levels, 14 of the 90 genes were selected as candidate dental pulp stem cell markers, particularly in relation to their multipotency characteristics. This characterization of cell clones obtained from a single specimen of human dental pulp provided information regarding new candidate marker genes for multipotent dental pulp stem cells, which could facilitate efficient analysis or enrichment of multipotent stem cells.

**Electronic supplementary material:**

The online version of this article (10.1007/s13577-020-00327-9) contains supplementary material, which is available to authorized users.

## Introduction

Mesenchymal stem cells (MSCs), have the capacity for clonogenic self-renewal, potential for multilineage differentiation (multipotency, including odontogenic, adipogenic, and chondrogenic differentiation) in vitro [1]; moreover, they exhibit tissue regeneration potential in vivo [2, 3]. Human postnatal MSCs have been identified in various tissues, including dental pulp; notably, dental pulp is an attractive cell source for regenerative therapy, because dental pulp tissues can be obtained from extracted teeth in a noninvasive manner, which are typically discarded.

However, populations of mesenchymal cells are often heterogeneous [4–6], such that they are composed of both genuine multipotent stem cells and committed progenitor cells with restricted differentiation potentials. This heterogeneity is a source of complexity that interferes with understanding of the stem cell mechanism. Because of the heterogeneity of MSCs, it remains controversial whether the multipotency of mesenchymal cell populations arises from genuine multipotent stem cells or the coexistence of distinct, committed progenitor cells. In previous attempts to address this issue, mesenchymal stem cell experiments have been performed with single cell-derived populations, which are regarded as clonal populations [7]. Multiple investigations have been performed to analyze human mesenchymal cell clones derived from tissues such as dental pulp [4, 6, 8–10]. However, in studies that involved small numbers of clones obtained from multiple donors, differences in gene expression among clones obtained from multiple donors might have reflected the different genetic backgrounds of the donors, rather than phenotypic differences between multipotent stem cells and committed progenitor cells [11]. Analysis of gene expression profiles among clones obtained from a single donor may allow researchers to eliminate the differences in genetic backgrounds that are associated with the use of multiple donors [12].

Distinct markers that define genuine MSCs are not yet well-established [13, 14]. Minimum criteria to define mesenchymal stromal (stem) cells has been proposed that mesenchymal stromal cells must express endoglin (ENG, better known as CD105), 5′-nucleotidase ecto (NT5E, better known as CD73), and Thy-1 cell surface antigen (THY1, better known as CD90) [1]. Moreover, various surface molecules have been used as putative mesenchymal stem cell markers. However, these markers were originally used to identify other tissues, such as endothelium or hematopoietic stem cells [14], this overlap may cause confusion regarding the proper identification of mesenchymal stromal cells. The identification of unique mesenchymal stem cell markers will increase the efficiency of analysis and facilitate the enrichment of multipotent MSCs.

In the present study, we analyzed the proliferation and differentiation characteristics of single cell-derived clones that were obtained from a single specimen of human dental pulp, then characterized the gene expression profiles of representative clones. We analyzed genes that demonstrated altered expression among clones with variations in differentiation potential.

## Materials and methods

### Cells and culture medium

A normal, impacted third molar was obtained from an 11-year-old female patient at the Nippon Dental University Hospital in Tokyo, Japan, with the approval by the Committee of Ethics at the Nippon Dental University School of Life Dentistry at Tokyo. Dental pulp tissue was separated from the tooth and dental pulp cell (DPC) populations were enzymatically released from the tissue [2, 15]. The culture medium used for cellular growth was minimum essential medium alpha (MEMα) (Thermo Fisher Scientific, MA, USA), supplemented with 20% fetal bovine serum (FBS) (SAFC Biosciences, KS, USA), 100 µM l-ascorbic acid phosphate magnesium salt *n*-hydrate (ascorbic acid) (Wako Pure Chemical, Osaka, Japan), 2 mM l-glutamine (Thermo Fisher Scientific), 100 units/ml penicillin and 100 µg/ml streptomycin (Thermo Fisher Scientific). The population doubling level (PDL) number was calculated by the formula, 2^*n*^ = *N*, where *n* = PDL and *N* = number of cells per flask. Mycoplasma tests of this DPC population were performed by JCRB Cell Bank, National Institutes Biomedical Innovation, Health and Nutrition (Osaka, Japan). Mycoplasma was not detected in the DPC population by PCR-based assay and by DNA fluorescence staining using VERO cells of JCRB Cell Bank.

### Isolation and culture of cell clones

Single cell-derived clones were isolated from DPC populations that were obtained from a single specimen of human dental pulp. DPC populations in primary culture (passage 0) were plated on 100-mm dishes (Corning, NY, USA) at a density of 200 cells/dish and incubated for 10 days to form colonies. Colonies with > 50 cells obviously distinct from other colonies were isolated separately, using penicillin cups with an inside diameter of 6 mm as cloning cylinders. Each clone was identified by clone (CL) number. All harvested clones were separately passaged.

### Flow cytometry

DPC populations were incubated with antibodies for 10 min at 4 °C after incubation with FcR blocking reagent (Miltenyi Biotec, North Rhine-Westphalia, Germany). The antibodies used were indicated in Table 1. After cells had been washed, they were analyzed using Guava® easyCyte flow cytometer (Luminex, TX, USA) and FlowJo software (version 10.4.2) (Becton, Dickinson and Company, NJ, USA).

**Table 1:** Antibodies used for flow cytometry

Primary antibody			Isotype control		
Target	Clone	Label	Target	Clone	Label
human CD105	43A4E1	PE ^*1^	mouse IgG_1_	IS5-21F5	PE
human CD73	AD2	PE	mouse IgG_1_	IS5-21F5	PE
human CD90	DG3	PE	mouse IgG_1_	IS5-21F5	PE
human CD146	541-10B2	PE	mouse IgG_1_	IS5-21F5	PE
human CD45	5B1	PE	mouse IgG_2a_	S43.10	PE
human CD34	AC136	PE	mouse IgG_2a_	S43.10	PE
human CD14	REA599	PE	mouse IgG_2a_	S43.10	PE
human CD79	HM47	PE	mouse IgG_1_	IS5-21F5	PE
human HLA-DR	IS5-20C4	PE	mouse IgG_2a_	S43.10	PE
human STRO-1	STRO-1	*2	mouse IgM	IS5-20C4	PE

^*^1: *PE* phycoerythrin

^*^2: Anti-human STRO-1 antibody was labelled with PE-conjugated anti-mouse IgM secondary antibody (clone: REA979)

Anti-human STRO-1antibody was purchased by R&D Systems, MN, USA, and other antibodies were purchased by Miltenyi Biotec, North Rhine-Westphalia, Germany

### Histochemical staining

DPC populations and clonal cells were both incubated in standard growth medium until they reached confluence. Then, cells were incubated in differentiation induction media as follows. To assess odontogenic differentiation, cells were incubated with MEMα that was supplemented with 10% FBS, 100 µM ascorbic acid, 2 mM l-glutamine, 10 mM sodium β-glycerophosphate *n*-hydrate (Wako Pure Chemical), and 10 nM dexamethasone (Wako Pure Chemical) [15]. To assess adipogenic differentiation, cells were incubated with MEMα that was supplemented with 20% FBS, 0.5 mM 3-isobutyl 1-methylxanthine (Merck, Darmstadt, Germany), 0.5 µM hydrocortisone (Merck), 60 µM indomethacin (Merck), 100 µM ascorbic acid, and 2 mM l-glutamine [16]. To assess chondrogenic differentiation, cells were incubated in Dulbecco’s modified Eagle medium (Thermo Fisher Scientific), supplemented with 10% FBS, 10 µg/ml insulin–transferrin–selenium-X (Thermo Fisher Scientific), 5.35 µg/ml linoleic acid (Merck), 1.25 µg/ml bovine serum albumin (Merck), 2.6 µM dexamethasone, 35 µM ascorbic acid, and 10 ng/ml transforming growth factor beta-3 (R&D Systems, MN, USA) [17]. To achieve differentiation induction, cells were cultured for up to 3 weeks. Chondrogenic induction of DPC populations was performed using adherent cells as well as cell pellets that were prepared by centrifuging cells (2.5 × 10^5^) in 15-ml conical polystyrene tubes (Corning) at 190 g for 5 min. After cells had been incubated in differentiation induction media, they were washed with phosphate-buffered saline (PBS) (Nissui Pharmaceutical, Tokyo, Japan) and fixed with 4% paraformaldehyde (Wako Pure Chemical) in PBS. Cells were then stained with either 1% Alizarin Red S (Merck) for odontogenic differentiation, 0.18% Oil Red O (Merck) for adipogenic differentiation, or 1% Alcian blue (pH 1.0) (Merck) for chondrogenic differentiation. For cell pellet analysis, frozen sections (10 μm thick) were stained with 1% Alcian blue.

### mRNA expression

Total cellular RNA was isolated and reverse-transcribed using a method described previously [18]. Quantitative reverse transcription polymerase chain reaction (qRT-PCR) analyses were performed using TaqMan® gene expression assays in a StepOne Plus® RT-PCR system (Thermo Fisher Scientific). The following human genes were targeted: integrin binding sialoprotein (IBSP) as a marker of odontogenic differentiation, lipoprotein lipase (LPL) as a marker of adipogenic differentiation, and collagen type X alpha 1 chain (COL10A1) as a marker of chondrogenic differentiation (assay IDs: Hs00173720_m1, Hs01012569_m1, and Hs00166657_m1, respectively; Thermo Fisher Scientific). The eukaryotic 18S rRNA gene (Thermo Fisher Scientific, catalogue number: 4319413E) was used as an endogenous control for expression. All experiments were performed in triplicate.

### Transplantation

DPC populations (approximately 2 × 10^6^) in primary culture (at 4.0 PDL) were mixed with 40 mg hydroxyapatite/tricalcium phosphate (HA/TCP) ceramic powder (Kobayashi Medical, Osaka, Japan) and incubated for 90 min at 37 °C. After cells had been centrifuged at 440 g for 7 min, the resulting cell pellets with HA/TCP were transplanted into immunocompromised beige mice (Crl: NIH-Lyst^bg^Foxn1^nu^Btk^xid^) (Charles River Laboratories Japan, Kanagawa, Japan), using a method described previously [15]. These experiments were performed under the approval by the Animal Experiments Committee of The Nippon Dental University School of Life Dentistry at Tokyo. Transplants were harvested from the immunocompromised mice and fixed, then decalcified with buffered 10% ethylenediaminetetraacetic acid (pH 8.0). Paraffin-embedded sections (5 µm thick) were stained with hematoxylin and eosin as described previously [2].

### Immunocytochemical staining

After cells were fixed and washed, cells were incubated with mouse anti-human STRO-1 antibody (MAB1038; R&D systems, 10 µg/ml) or mouse anti-human CD146 antibody (NCL-CD146; Leica Biosystems, Baden-Württemberg, Germany, 1:25 dilution) and stained with Histostain®-SP kit (AEC, broad spectrum; Thermo Fisher Scientific), in accordance with the manufacturers’ instructions. Mouse anti-human isotype control (Thermo Fisher Scientific) was used as a negative control. The detection criteria were as follows: at least one positive cell detected in three visual fields with × 20 magnification was classified as a positive finding, while a lack of positive cells was classified as a negative finding.

### Analysis of gene expression profiles

DNA microarray analyses of representative clones were performed to compare the gene expression profiles of genes related to multipotency. After total cellular RNA from each clone had been isolated and reverse-transcribed, DNA microarray analyses were performed by Cell Innovator Inc. (Fukuoka, Japan), using Affymetrix GeneChip® Human Gene 1.0 ST Arrays (Affymetrix, Thermo Fisher Scientific). Raw data were processed for gene-level analysis with median polish summarisation and quantile normalisation by Affymetrix® Expression Console™ 1.1 software (Thermo Fisher Scientific) to obtain normalised intensity values. The expression ratio between clones was calculated from the signal intensity values of each probeset. Unsupervised hierarchical clustering analyses, which are represented in heat maps of the signal intensity values, were performed with Multiple Experiment Viewer software [19]. The databases of Ingenuity Pathway Analysis (IPA) (Ingenuity, QIAGEN, North Rhine-Westphalia, Germany) and Gene Ontology [20] and the scientific literature were used to compile lists of ‘stemness’- or ‘differentiation’-related genes (Supplemental Tables S2, S3).

## Results

### Differentiation potentials and tissue regeneration potentials of dental pulp cell populations

**Fig. 1:**
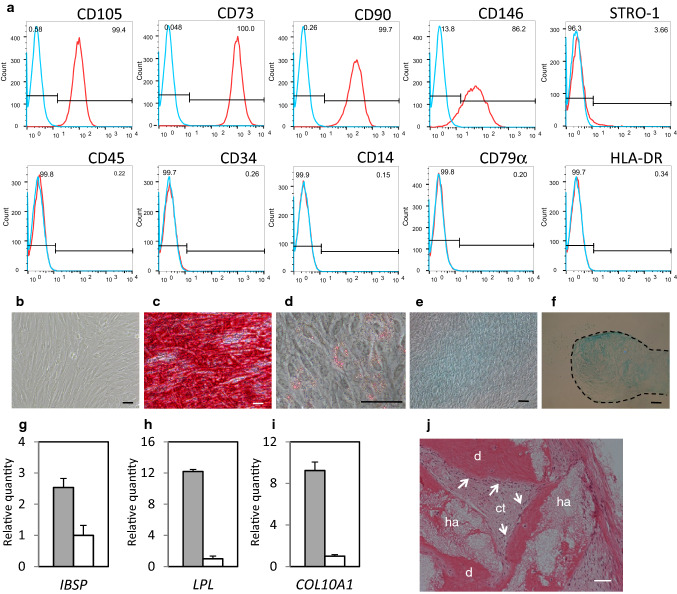
Differentiation potentials and tissue regeneration characteristics of human dental pulp cell populations. **a** Expression characteristics of cell surface molecules of dental pulp cell populations at 17.8 PDL analyzed by flow cytometry. **b** Cell morphologies of dental pulp cell populations at 4.0 PDL. **c** Alizarin Red S staining of dental pulp cell populations cultured in odontogenic differentiation medium for 21 days. **d** Oil Red O staining of dental pulp cell populations cultured in adipogenic differentiation medium for 8 days. **e**, **f** Alcian blue staining of dental pulp cell populations cultured in chondrogenic differentiation medium. **e** Adherent cells after 8 days of induction. **f** Cell pellet after 21 days of induction. The border of the pellet is indicated with a dashed line. **g**–**i** Gene expression levels of differentiation marker genes in each differentiated dental pulp cell population, analyzed by qRT-PCR. Grey bar: differentiation-induced cells; white bar: control cells. *n* = 3. Data are shown as mean (standard deviation). **g** Expression of IBSP for cells cultured in odontogenic differentiation medium. **h** Expression of LPL for cells cultured in adipogenic differentiation medium. **i** Expression of COL10A1 for cells cultured in chondrogenic differentiation medium. **j** Hematoxylin and eosin-stained section of regenerated dentin/pulp-like complex tissues 3 months after transplantation of dental pulp cell populations with HA/TCP into immunocompromised mice. *d* dentin-like structure, *ct* connective tissue; arrows: odontoblast-like cells, *ha* HA/TCP carriers. Scale bars in (**b**–**f**, **j**) = 50 μm. *qRT-PCR* quantitative reverse transcription polymerase chain reaction, *PDL* population doubling level, *HA/TCP* hydroxyapatite/tricalcium phosphate

We first investigated the multipotency of heterogeneous human DPC populations in vitro and in vivo. DPC populations obtained from a single specimen of human dental pulp expressed CD105, CD73, CD90, CD146, and (weakly) STRO-1, whereas they lacked expression of hemocyte-associated markers (CD45, CD34, CD14, CD79, and HLA-DR) (Fig. 1a). The human DPC populations exhibited fibroblast-like morphology in vitro (Fig. 1b). Differentiation-induced DPC populations were positively stained with Alizarin Red S (odontogenic differentiation) (Fig. 1c), Oil Red O (adipogenic differentiation) (Fig. 1d), and Alcian blue (chondrogenic differentiation) (Fig. 1e, f). The expression levels of IBSP*,* LPL, and COL10A1 (respective odontogenic, adipogenic, and chondrogenic differentiation markers) were considerably greater in differentiated cell populations than in undifferentiated control populations (Fig. 1g–i). Dentin/pulp-like complex tissues were formed after transplantation of human DPC populations into immunocompromised mice (Fig. 1j). Odontoblast-like cells were observed in connective tissue adjacent to the surface of the dentin-like structures (Fig. 1j). These findings demonstrated that heterogeneous human DPC populations exhibit multipotency in vitro and tissue regeneration potential in vivo.

### Colony-picking and proliferation of isolated clones

Colony-forming single cell-derived clones were isolated from heterogeneous multipotent human DPC populations. The single cell ratio of the cell suspension at the time of plating was > 97%. The colony formation rate was 64.3 ± 3.01%. Fifty colonies (clones) (CL 1–CL 50) were isolated and separately cultured until growth cessation. The PDL at growth cessation varied among clones, from 30.1 PDL to 67.3 PDL (Supplemental Table S1).

### Expression of surface markers by each clone

**Fig. 2:**
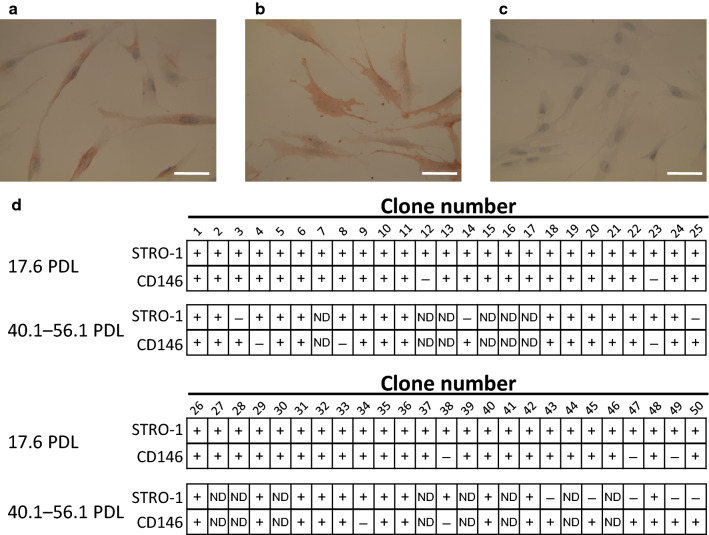
Expression characteristics of surface markers by each clone. **a**–**c** Representative immunocytochemical stainings of clones. Scale bars = 50 μm. (**a**) STRO-1-positive, (**b**) CD146-positive, and (**c**) negative control. (**d**) STRO-1 and CD146 expression in each clone at 17.6 PDL and 40.1–56.1 PDL. +  positive expression, − negative expression. Some clones were not tested at > 40 PDL, because they stopped proliferating prior to 40 PDL (indicated as ‘ND’, not determined)

The expression of two well-known mesenchymal stem cell surface markers (STRO-1 and CD146) by each clone was examined by immunocytochemical analysis (Fig. 2). Forty-five (90%) of the 50 clones were positive for both STRO-1 and CD146 expression at 17.6 PDL. Thirty-six of the 50 clones were examined at both 17.6 PDL and > 40 PDL. Twenty-three of these 36 clones (64%) were positive for STRO-1 and CD146 expression at both 17.6 PDL and > 40 PDL, demonstrating that the majority of clones maintained expression of both mesenchymal stem cell surface markers throughout long-term culture.

### Differentiation potentials of each clone

**Fig. 3:**
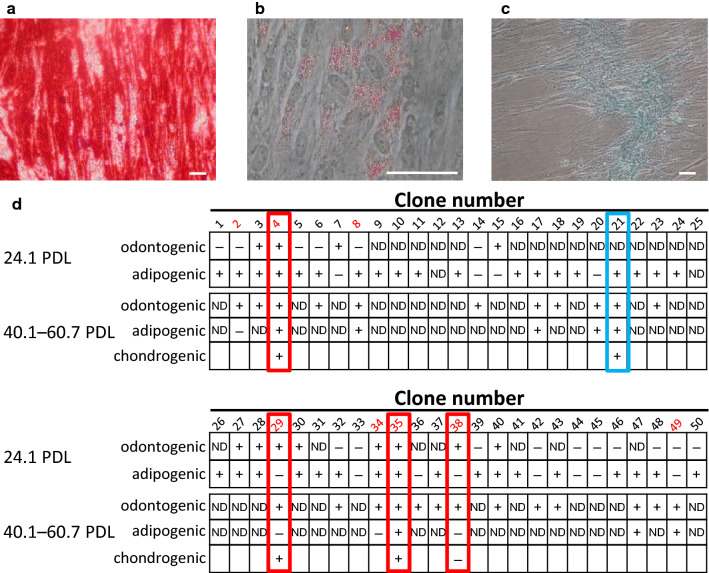
Differentiation potentials of each clone. **a**–**c** Representative histochemical stainings of clones. Scale bars = 50 μm. **a** Alizarin Red S staining for odontogenic differentiation. **b** Oil Red O staining for adipogenic differentiation. **c** Alcian blue staining for chondrogenic differentiation (adherent cells). **d** Odontogenic, adipogenic, and chondrogenic differentiation potentials of each clone at 24.1 PDL and 40.1–60.7 PDL. Eight clones were assayed for odontogenic and adipogenic differentiation potentials at both 24.1 PDL and > 40 PDL (clone numbers indicated in red). Four clones (indicated by red rectangles) maintained their differentiation potentials during long-term culture; CL 21 (indicated by a blue rectangle) was the clone with the highest proliferation rate. +  positive, − negative. Some clones were not tested, because they detached from the culture surface (possibly because of overgrowth) during the differentiation period at 24.1 PDL. In differentiation analysis at > 40 PDL, some clones were not assayed, because they stopped proliferating prior to 40 PDL. Some clones exhibited positive staining for Alizarin Red S and/or Oil Red O under the culture conditions with control medium at 40.1–60.7 PDL. The differentiation potentials of these spontaneously differentiated clones were recorded as ‘undetermined’. In the table, ‘ND’ (not determined) indicates clones that detached or stopped proliferation, or spontaneously differentiated

We examined the odontogenic and adipogenic differentiation potentials of each clone at early (24.1 PDL) and late (> 40 PDL) stages of culture (Fig. 3). Odontogenic and adipogenic differentiation potentials at 24.1 PDL were analyzed in 28 clones; eight of these 28 clones (29%) were both odontogenic and adipogenic, four clones (14%) were odontogenic only, 10 clones (36%) were adipogenic only, and six clones (21%) did not demonstrate either differentiation potential.

In total eight clones were assayed for odontogenic and adipogenic differentiation potentials at both 24.1 PDL and > 40 PDL (Fig. 3). Four of these eight clones exhibited similar differentiation potentials at 24.1 PDL and > 40 PDL, suggesting that their differentiation potentials were maintained throughout long-term culture (CL 4 and CL 35, both odontogenic and adipogenic; CL29 and CL 38, odontogenic only). Notably, the odontogenic differentiation potential of CL 21 was not assayed at 24.1 PDL, because these cells detached during the differentiation period; however, this clone maintained adipogenic differentiation potential and exhibited odontogenic differentiation at 60.7 PDL. Furthermore, CL 21 demonstrated the highest proliferation ability (Supplemental Table S1). We identified five representative clones and tested their chondrogenic differentiation potentials at > 40 PDL: CL 4, CL 21, CL 29, CL 35, and CL 38 (Fig. 3). All of these clones, with the exception of CL 38, exhibited chondrogenic differentiation potential.

A summary of the differentiation potentials among representative clones is shown in Table 2. CL 4, CL 21, and CL 35 exhibited tri-lineage differentiation potential (tripotent). CL 29 and CL 38 exhibited bi-lineage and uni-lineage differentiation potentials, respectively (bipotent and unipotent, respectively). All representative clones maintained the expression of STRO-1 (Fig. 2). However, CL 4 had lost expression of CD146 by 43.7 PDL, while CL 38 did not exhibit expression of CD146 throughout the experiment (Fig. 2).

**Table 2:** Clones with a variety of differentiation potentials

Clone no.	Differentiation potential				
	Tripotent			Bipotent	Unipotent
	CL 4	CL 21	CL 35	CL 29	CL 38
Odontogenic	+	+	+	+	+
Chondrogenic	+	+	+	+	−
Adipogenic	+	+	+	−	−

*CL* clone

### Gene expression profiles of representative clones

**Fig. 4:**
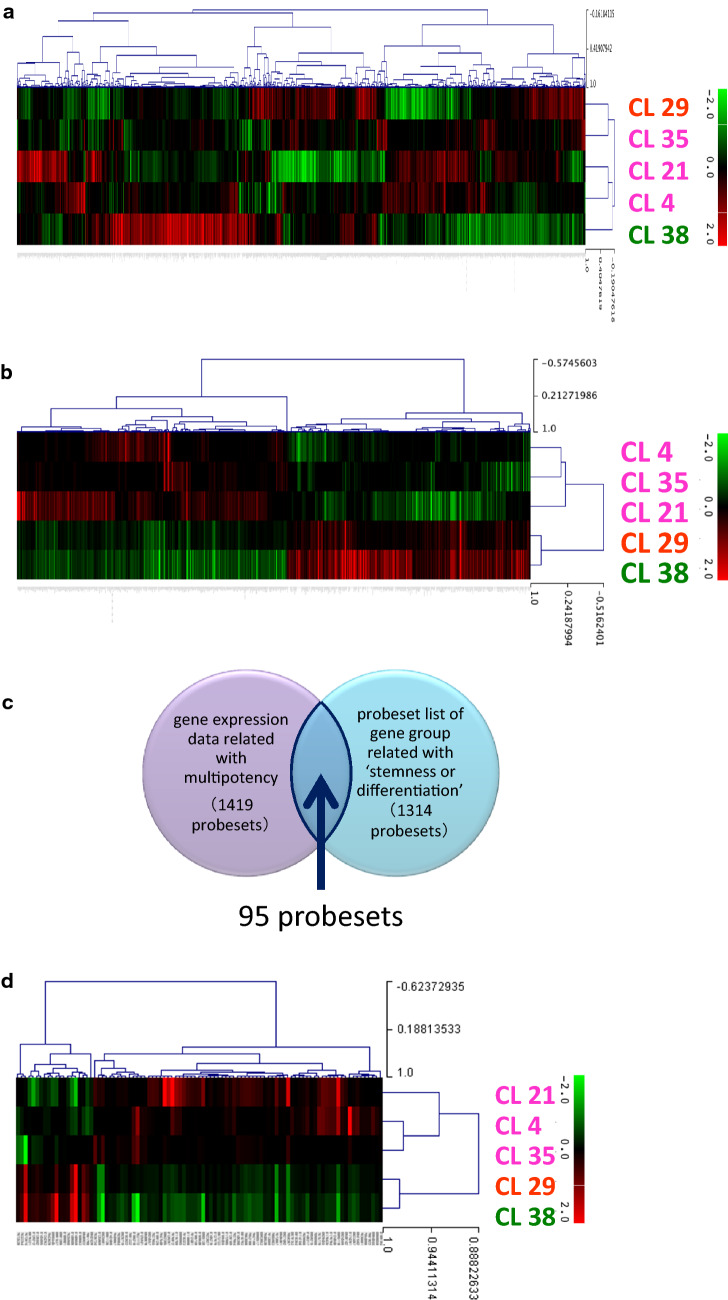
Gene expression profiles of representative clones. **a** Unsupervised hierarchical clustering heat map of genes with altered expression in five representative clones. **b** Unsupervised hierarchical clustering heat map of genes with altered expression correlating positively or negatively with multipotency. **c** Venn diagram representing the strategy for analysis of gene expression profiles related to multipotency and ‘stemness or differentiation’. **d** Unsupervised hierarchical clustering heat map of 95 probesets related to multipotency and ‘stemness or differentiation’

Gene expression analyses were performed on the above-selected five representative clones to detect genes related to multipotency. Total RNA of representative clones were collected from 24.1 to 29.1 PDL. We compared data sets of gene expression profiles among the five clones. The criteria for identification as a gene with altered expression were that the probeset signal intensity value was > 100 and the ratio of the signal intensity value for each comparison was > 1.5 or < 0.67 in at least one comparison. In total, 1950 probesets met these criteria. Unsupervised hierarchical clustering analyses of the 1950 probesets, utilising genes with altered expression between clones (represented by heat mapping), revealed that the clusters were not aligned on the basis of multipotency (Fig. 4a). This analysis demonstrated that major genes with altered expression were not directly related to multipotency.

Then we used an alternative analysis method for detection of genes related to multipotency. We identified probesets of genes that were correlated either positively or negatively with multipotency. Genes that were correlated positively with multipotency were *tri*- > *bi*- > unipotent (CL 4 > CL 29 > CL 38; CL 21 > CL 29 > CL 38; or CL 35 > CL 29 > CL 38) (808 probesets, corresponding to 754 genes). In contrast, genes that were correlated negatively with multipotency were uni- > *bi*- > *tri*-potent (CL 38 > CL 29 > CL 4; CL 38 > CL 29 > CL 21; or CL 38 > CL 29 > CL 35) (611 probesets, corresponding to 473 genes). Hence, 1419 (808 + 611) probesets were correlated with multipotency, corresponding to 1227 (754 + 473) genes. Unsupervised hierarchical clustering analyses of genes with altered expression that were correlated positively or negatively with multipotency, as represented by heat mapping, are shown in Fig. 4b.

Additionally, a list of genes related to ‘stemness or differentiation’ was constructed using information from gene databases (IPA and Gene Ontology) and the scientific literature; it consisted of 1314 probesets, corresponding to 1246 genes (Supplemental Tables S2, S3). We reduced the number of candidate dental pulp stem cell marker genes based on overlap between genes related to multipotency (1419 probesets of 1227 genes from DNA microarray data) and ‘stemness or differentiation’ (1314 probesets of 1246 genes from databases and the literature) (Fig. 4c). The list of genes that were correlated with multipotency in present experiment partially overlapped with the list of genes related to ‘stemness or differentiation’ constructed using information from databases and the literature, suggesting that the genes correlated with multipotency were also related to ‘stemness or differentiation’. There were 95 overlapping probesets, which corresponded to 90 distinct genes (Fig. 4d; Supplemental Table S4). From among these 90 genes, we selected 14 representative genes, shown in Table 3, based on large changes in expression levels and a predicted location (either plasma membrane or extracellular space) for the expressed protein product that would facilitate its detection by flow cytometry or immunocytochemical analysis. Nine of these genes were positively correlated with multipotency, while five of these genes were negatively correlated with multipotency. Thus, these 14 genes are related to both multipotency and ‘stemness or differentiation’, and are candidates for use as markers of multipotent mesenchymal stem cells.

**Table 3:** Fourteen representative genes related to multipotency and ‘stemness or differentiation’

Symbol	Gene name
Genes positively correlated with multipotency	
* ATP8B1*	ATPase phospholipid transporting 8B1
* DSP*	Desmoplakin
* ICAM1*	Intercellular adhesion molecule 1
* INHBA*	Inhibin beta A subunit
* NNAT*	Neuronatin
* OXTR*	Oxytocin receptor
* SERPINE1*	Serpin family E member 1
* SORT1*	Sortilin 1
* SRGN*	Serglycin
Genes negatively correlated with multipotency	
* ADGRA2*	Adhesion G protein-coupled receptor A2
* ANTXR1*	Anthrax toxin receptor 1
* COL1A2*	Collagen type I alpha 2 chain
* COL3A1*	Collagen type III alpha 1 chain
* ITGA8*	Integrin subunit alpha 8

## Discussion

The present investigation demonstrated that colony-forming single cell-derived clones, which are obtained from single dental pulp, varied in proliferation ability, surface marker expression, differentiation potential, and gene expression. Importantly, a single specimen of dental pulp contained both multipotent stem cell-like clones and progenitor-like clones with restricted differentiation potentials. These results support the findings of previous reports regarding variation in single cell-derived clones [9, 12, 21–26].

The clonogenic cells in this study expressed both STRO-1 and CD146 at a high frequency at 17.6 PDL (Fig. 2d). However, some isolated clonogenic clones positive for STRO-1 or CD146 exhibited restricted differentiation potentials. Gharibi and Hughes analyzed the expression of stem cell surface markers by flow cytometry; they showed that the expression of CD146 and other stem cell markers persisted despite the loss of differentiation potentials during long-term culture [27]. Therefore, cells expressing stem cell markers may include cells with restricted differentiation potentials.

Somoza et al. analyzed 38 human bone marrow-derived cell clones and found that 10 (26%) were both osteogenic and adipogenic, two (5%) were osteogenic only, 21 (55%) were adipogenic only, and five (13%) did not demonstrate either differentiation potential [23], notably, these results in bone marrow-derived cell clones were similar to our results in dental pulp-derived cell clones (Fig. 3 at 24.1 PDL).

In an additional study, Muraglia et al. analyzed the hierarchy of multipotency (osteogenic, chondrogenic, and adipogenic differentiation) in human bone marrow-derived cell clones. They reported that clones progressively lost adipogenic differentiation potential, then lost chondrogenic differentiation potential with an increasing number of cell doublings [24]. This hierarchy was also present in our results (Table 2).

A portion of our 90 selected genes overlapped with those described in other reports of gene expression profiles in MSCs. Mareddy et al. compared fast-growing and slow-growing clones from three donors; they identified 17 upregulated and eight downregulated genes in fast-growing clones, compared with slow-growing clones [25]. Two of the selected 90 genes in our study were consistent with their findings [bone morphogenetic protein 2 (BMP2) and delta like canonical Notch ligand 3 (DLL3)]. In another study, Menicanin et al. compared clones that exhibited high growth/multi-differentiation potentials with clones that exhibited low growth potentials; they identified 24 genes that were upregulated in clones that exhibited high growth/multi-differentiation potentials [4]. Notably, replication protein A3 (RPA3) was identified in both their study and our study. In yet another investigation, Sworder et al. measured tissue regeneration potentials in clones and identified 19 genes that were differentially expressed in multipotent clones [12], including two genes [BMP2 and intercellular adhesion molecule 1 (ICAM1)] that were also identified in our study. In all three of these prior studies, the investigators used bone marrow-derived mesenchymal cells. Accordingly, we found some overlap in the data, but observed multiple differences that may be related to the human dental pulp origin of our cells. Additional, detailed experiments are thus required to elucidate differences between mesenchymal cells derived from bone marrow and those derived from dental pulp.

We selected 14 genes (Table 3) for further analysis from among the 90 genes that were related to both multipotency and ‘stemness or differentiation’. One of these, desmoplakin (DSP) is expressed in odontoblasts and cultured dental pulp fibroblasts [28]. ICAM1 is reportedly indispensable for MSC-mediated immunosuppression [29, 30]. Serpin family E member 1 (SERPINE1) has been reported as an adipogenesis-related gene [31]. Another gene, sortilin 1 (SORT1) has been reported as an adipogenesis- and osteogenesis-related gene [31]. Collagen type I alpha 2 chain (COL1A2) has been reported as an osteogenesis-related gene [31]. Collagen type III alpha 1 chain (COL3A1) is expressed various oral mesenchymal stem cell populations in vitro [3], and has been reported as a marker for odontoblast differentiation [17]. ATPase phospholipid transporting 8B1 (ATP8B1), ICAM1, adhesion G protein-coupled receptor A2 (ADGRA2), and anthrax toxin receptor 1 (ANTXR1) were reported as stemness-related genes that were downregulated during differentiation but upregulated during dedifferentiation in MSCs [32]. In addition, oxytocin receptor (OXTR) and serglycin (SRGN) are upregulated during osteoblast differentiation [31]. These candidate markers are expected to be useful tools to isolate or enrich genuine multipotent dental pulp stem cells for clinical regeneration therapies. In the case of clinical use of dental pulp stem cells for immunomodulatory features or secreted factors [33], other tools might be needed, because candidate markers in the present study were selected in relation to multipotency.

Variations in differentiation potential and gene expression among clones obtained from a single specimen of dental pulp were analyzed in this study. The advantage of this strategy was that the underlying genetic variance was minimised among clones. Thus, our strategy allowed clearer detection of differences in gene expression among clones that exhibit disparate differentiation potentials. However, Sworder et al. analyzed clones obtained from a single donor and demonstrated that expression level of one of their candidate marker genes might vary among donors [12]. Our planned future studies include the analysis of a large series of cells from multiple donors to confirm the most reliable markers among our candidate genes for the identification of genuine multipotent dental pulp stem cells.

In present study, fifty clones were isolated from a single specimen of human dental pulp. We assessed their proliferation abilities, surface cell marker expression patterns, and differentiation potentials. Analysis of the gene expression profiles of five representative clones enabled identification of 14 genes related to multipotency and ‘stemness or differentiation,’ as candidate markers for dental pulp stem cells. These candidate genes could be used to isolate and manipulate multipotent dental pulp stem cells for regeneration therapies.

## Electronic supplementary material

Below is the link to the electronic supplementary material.

Supplementary file1 (PDF 32 kb)

Supplementary file2 (PDF 73 kb)

Supplementary file3 (PDF 10 kb)

Supplementary file4 (PDF 18 kb)
