# Supplementary material for: Characterization of proliferation, differentiation potential, and gene expression among clonal cultures of human dental pulp cells
Source: Hum Cell. 2020 Mar 16;33(3):490–501. doi: 10.1007/s13577-020-00327-9 (PMC7324427; doi:10.1007/s13577-020-00327-9)
Supplement: Supplementary file 1 — Supplementary file1 (PDF 32 kb) [file 13577_2020_327_MOESM1_ESM.pdf]

Supplemental Table S1. Length of culture (days) and population doubling level (PDL) at growth cessation for each clone (CL).

clone	culture period (days)	PDL at growth cessation
CL 1	207	48.1
CL 2	207	55.9
CL 3	175	44.7
CL 4	207	56.7
CL 5	207	48.7
CL 6	175	46.7
CL 7	175	38.1
CL 8	207	58.7
CL 9	235	49.1
CL 10	235	57.1
CL 11	235	45.1
CL 12	175	37.1
CL 13	175	30.1
CL 14	235	50.7
CL 15	175	38.7
CL 16	207	33.1
CL 17	175	40.1
CL 18	175	49.7
CL 19	175	47.7
CL 20	207	44.7
CL 21	255	67.3
CL 22	206	44.1
CL 23	207	53.9
CL 24	207	48.1
CL 25	206	43.7
CL 26	255	52.1
CL 27	207	33.1
CL 28	207	35.1
CL 29	207	55.7
CL 30	207	35.1
CL 31	207	47.7
CL 32	255	52.1
CL 33	207	43.7
CL 34	207	49.7
CL 35	207	46.9
CL 36	207	49.3
CL 37	207	41.1
CL 38	207	53.7
CL 39	207	41.1
CL 40	207	46.1
CL 41	207	34.1
CL 42	255	48.7
CL 43	207	49.1
CL 44	207	33.3
CL 45	207	42.7
CL 46	207	38.1
CL 47	207	57.0
CL 48	207	43.1
CL 49	207	51.3
CL 50	207	50.7

characterization of proliferation, differentiation potential, and gene expression among clonal cultures of human dental pulp cells

Tomoko Kobayashi[1,2], Daisuke Torii [3], Takanori Iwata [2], Yuichi Izumi [2,4], Masanori Nasu [1], Takeo W. Tsutsui[3]

[1] Research Center for Odontology, School of Life Dentistry at Tokyo, The Nippon Dental University

[2] Department of Periodontology, Graduate School of Medical and Dental Sciences, Tokyo Medical and Dental University (TMDU)

[3] Department of Pharmacology, School of Life Dentistry at Tokyo, The Nippon Dental University

[4] Oral Care Perio Center, Southern TOHOKU General Hospital, Southern TOHOKU Research Institute for Neuroscience

Corresponding author: Takeo W. Tsutsui (ryuryu@tky.ndu.ac.jp)
