# Supplementary material for: Characterization of proliferation, differentiation potential, and gene expression among clonal cultures of human dental pulp cells
Source: Hum Cell. 2020 Mar 16;33(3):490–501. doi: 10.1007/s13577-020-00327-9 (PMC7324427; doi:10.1007/s13577-020-00327-9)
Supplement: Supplementary file 2 — Supplementary file2 (PDF 73 kb) [file 13577_2020_327_MOESM2_ESM.pdf]

characterization of proliferation, differentiation potential, and gene expression among clonal cultures of human dental pulp cells

Tomoko Kobayashi[1,2], Daisuke Torii [3], Takanori Iwata [2], Yuichi Izumi [2,4], Masanori Nasu [1], Takeo W. Tsutsui[3]

1 Research Center for Odontology, School of Life Dentistry at Tokyo, The Nippon Dental University
2 Department of Periodontology, Graduate School of Medical and Dental Sciences, Tokyo Medical and Dental University (TMDU)
3 Department of Pharmacology, School of Life Dentistry at Tokyo, The Nippon Dental University
4 Oral Care Perio Center, Southern TOHOKU General Hospital, Southern TOHOKU Research Institute for Neuroscience

Corresponding author: Takeo W. Tsutsui (ryuryu@tky.ndu.ac.jp)

Supplemental Table S2. Probeset list of gene groups related to 'stemness or differentiation'.
Genes were extracted from gene databases [Ingenuity Pathway Analysis (IPA) and Gene Ontology] and published scientific literature, then converted to probeset IDs.

Affymetrix_ ProbeSet_ID	Gene_Symbol	Gene_Description
7896952	ATAD3A	ATPase family, AAA domain containing 3A
7897068	SKI	v-ski sarcoma viral oncogene homolog (avian)
7897132	PRDM16	PR domain containing 16
7897280	HES3	hairy and enhancer of split 3 (Drosophila)
7897737	C1orf187	chromosome 1 open reading frame 187
7898537	PAX7	paired box 7
7898693	ALPL	alkaline phosphatase, liver/bone/kidney
7898739	CDC42	"cell division cycle 42 (GTP binding protein, 25kDa) "
7898799	C1QC	"complement component 1, q subcomponent, C chain "
7898988	CLIC4	chloride intracellular channel 4
7899167	LIN28A	lin-28 homolog A (C. elegans)
7899187	HMGN2	high-mobility group nucleosomal binding domain 2
7899265	SFN	stratifin
7899562	PTPRU	"protein tyrosine phosphatase, receptor type, U "
7899753	LCK	lymphocyte-specific protein tyrosine kinase
7899774	HDAC1	histone deacetylase 1
7899790	TSSK3	testis-specific serine kinase 3
7900146	ZC3H12A	zinc finger CCCH-type containing 12A
7900340	BMP8A	bone morphogenetic protein 8a
7900699	CDC20	cell division cycle 20 homolog (S. cerevisiae)
7900792	PTPRF	protein tyrosine phosphatase, receptor type, F
7901073	UROD	uroporphyrinogen decarboxylase
7901123	NASP	nuclear autoantigenic sperm protein (histone-binding)
7901140	MAST2	microtubule associated serine/threonine kinase 2
7901363	CDKN2C	"cyclin-dependent kinase inhibitor 2C (p18, inhibits CDK4) "
7901557	DMRTB1	"DMRT-like family B with proline-rich C-terminal, 1 "

7901696	PCSK9	proprotein convertase subtilisin/kexin type 9
7901913	FOXD3	forkhead box D3
7902227	GADD45A	growth arrest and DNA-damage-inducible, alpha
7902367	ACADM	acyl-CoA dehydrogenase, C-4 to C-12 straight chain
7903358	VCAM1	vascular cell adhesion molecule 1
7903461	NTNG1	netrin G1
7903786	CSF1	colony stimulating factor 1 (macrophage)
7903878	RBM15	RNA binding motif protein 15
7904025	WNT2B	"wingless-type MMTV integration site family, member 2B "
7904702	NOTCH2NL	Notch homolog 2 (Drosophila) N-terminal like
7904726	TXNIP	thioredoxin interacting protein
7905220	ECM1	extracellular matrix protein 1
7905395	PSMB4	proteasome (prosome, macropain) subunit, beta type, 4
7905544	SPRR1A	small proline-rich protein 1A
7905789	IL6R	interleukin 6 receptor
7905854	ZBTB7B	zinc finger and BTB domain containing 7B
7905938	RAG1AP1	recombination activating gene 1 activating protein 1
7906061	SYT11	synaptotagmin XI
7906107	SEMA4A	"sema domain, immunoglobulin domain (Ig), transmembrane domain (TM) and short cytoplasmic domain, (semaphorin) 4A "
7906140	BGLAP	bone gamma-carboxyglutamate (gla) protein
7906244	NTRK1	"neurotrophic tyrosine kinase, receptor, type 1 "
7906400	IFI16	"interferon, gamma-inducible protein 16 "
7906954	PBX1	pre-B-cell leukemia homeobox 1
7907160	ATP1B1	ATPase, Na+/K+ transporting, beta 1 polypeptide
7907702	SOAT1	sterol O-acyltransferase 1
7908409	RGS2	"regulator of G-protein signaling 2, 24kDa "
7908694	NAV1	neuron navigator 1
7908917	BTG2	"BTG family, member 2 "
7909027	NFASC	neurofascin
7909225	DYRK3	dual-specificity tyrosine-(Y)-phosphorylation regulated kinase 3
7909332	CD55	CD55 molecule, decay accelerating factor for complement (Cromer blood group)
7909628	FLVCR1	feline leukemia virus subgroup C cellular receptor 1
7909708	CENPF	"centromere protein F, 350/400ka (mitosin) "
7909789	TGFB2	"transforming growth factor, beta 2 "
7909890	HLX	H2.0-like homeobox
7910134	MIXL1	Mix1 homeobox-like 1 (Xenopus laevis)
7910146	PSEN2	presenilin 2 (Alzheimer disease 4)
7910217	WNT3A	"wingless-type MMTV integration site family, member 3A "
7910265	OBSCN	"obscurin, cytoskeletal calmodulin and titin-interacting RhoGEF "
7910550	TSNAX	translin-associated factor X
7910694	EDARADD	EDAR-associated death domain
7911376	HES4	hairy and enhancer of split 4 (Drosophila)
7911422	SDF4	stromal cell derived factor 4

7911506	DVL1	dishevelled, dsh homolog 1 (Drosophila)
7911750	HES5	hairy and enhancer of split 5 (Drosophila)
7911989	RPL22	ribosomal protein L22
7912012	ACOT7	acyl-CoA thioesterase 7
7912157	ERRFI1	ERBB receptor feedback inhibitor 1
7912481	MAD2L2	MAD2 mitotic arrest deficient-like 2 (yeast)
7912706	EPHA2	EPH receptor A2
7913450	HSPG2	heparan sulfate proteoglycan 2
7913547	WNT4	"wingless-type MMTV integration site family, member 4 "
7913655	ID3	"inhibitor of DNA binding 3, dominant negative helix-loop-helix protein "
7913858	PAQR7	progestin and adipoQ receptor family member VII
7913864	STMN1	stathmin 1
7913869	STMN1	stathmin 1
7914021	SLC9A1	"solute carrier family 9 (sodium/hydrogen exchanger), member 1 "
7914433	BAI2	brain-specific angiogenesis inhibitor 2
7915156	POU3F1	POU class 3 homeobox 1
7915252	BMP8B	bone morphogenetic protein 8b
7915614	PTCH2	patched 2
7915955	SPATA6	spermatogenesis associated 6
7915991	DMRTA2	DMRT-like family A2
7916112	RAB3B	RAB3B, member RAS oncogene family
7916185	ZCCHC11	"zinc finger, CCHC domain containing 11 "
7916541	DAB1	disabled homolog 1 (Drosophila)
7916562	HNRNPA1	heterogeneous nuclear ribonucleoprotein A1
7916609	JUN	jun oncogene
7916669	DOCK7	dedicator of cytokinesis 7
7917182	ELTD1	EGF, latrophilin and seven transmembrane domain containing 1
7917199	TTLL7	"tubulin tyrosine ligase-like family, member 7 "
7917532	GBP2	guanylate binding protein 2, interferon-inducible
7917599	BARHL2	BarH-like homeobox 2
7917649	TGFBR3	"transforming growth factor, beta receptor III "
7917676	GLMN	"glomulin, FKBP associated protein "
7918323	SORT1	sortilin 1
7918359	AMIGO1	adhesion molecule with Ig-like domain 1
7918813	NRAS	neuroblastoma RAS viral (v-ras) oncogene homolog
7918857	TSPAN2	tetraspanin 2
7918869	NGF	nerve growth factor (beta polypeptide)
7919095	NOTCH2	Notch homolog 2 (Drosophila)
7919751	MCL1	myeloid cell leukemia sequence 1 (BCL2-related)
7919815	CTSK	cathepsin K
7919825	ARNT	aryl hydrocarbon receptor nuclear translocator
7919898	SEMA6C	"sema domain, transmembrane domain (TM), and cytoplasmic domain, (semaphorin) 6C "
7920165	FLG	filaggrin

7920217	SPRR2G	small proline-rich protein 2G
7920472	TPM3	tropomyosin 3
7920575	PBXIP1	pre-B-cell leukemia homeobox interacting protein 1
7920839	RIT1	Ras-like without CAAX 1
7920984	CCT3	chaperonin containing TCP1, subunit 3 (gamma)
7921014	MEF2D	myocyte enhancer factor 2D
7921099	CRABP2	cellular retinoic acid binding protein 2
7921133	HDGF	hepatoma-derived growth factor
7921144	SH2D2A	SH2 domain containing 2A
7921538	IGSF8	immunoglobulin superfamily, member 8
7921936	LMX1A	"LIM homeobox transcription factor 1, alpha "
7922432	RC3H1	ring finger and CCCH-type zinc finger domains 1
7922976	PTGS2	prostaglandin-endoperoxide synthase 2 (prostaglandin G/H synthase and cyclooxygenase)
7923027	GLRX2	glutaredoxin 2
7923086	ASPM	"asp (abnormal spindle) homolog, microcephaly associated (Drosophila) "
7923528	MYOG	myogenin (myogenic factor 4)
7924029	LAMB3	"laminin, beta 3 "
7924058	IRF6	interferon regulatory factor 6
7924832	WNT9A	wingless-type MMTV integration site family, member 9A
7924996	C1orf198	chromosome 1 open reading frame 198
7925565	HNRNPU	heterogeneous nuclear ribonucleoprotein U (scaffold attachment factor A)
7926105	GATA3	GATA binding protein 3
7926319	SUV39H2	suppressor of variegation 3-9 homolog 2 (Drosophila)
7926368	VIM	vimentin
7926609	BMI1	BMI1 polycomb ring finger oncogene
7926851	WAC	WW domain containing adaptor with coiled-coil
7926916	ZEB1	zinc finger E-box binding homeobox 1
7927389	MAPK8	mitogen-activated protein kinase 8
7927631	DKK1	dickkopf homolog 1 (Xenopus laevis)
7927710	CDK1	cyclin-dependent kinase 1
7927814	SIRT1	sirtuin (silent mating type information regulation 2 homolog) 1 (S. cerevisiae)
7927955	KIAA1279	KIAA1279
7927964	SRGN	serglycin
7928069	COL13A1	"collagen, type XIII, alpha 1 "
7928308	DDIT4	DNA-damage-inducible transcript 4
7928855	BMPR1A	"bone morphogenetic protein receptor, type IA "
7928937	MINPP1	multiple inositol-polyphosphate phosphatase 1
7929026	ACTA2	actin, alpha 2, smooth muscle, aorta
7929282	HHEX	hematopoietically expressed homeobox
7929334	CEP55	centrosomal protein 55kDa
7929744	NKX2-3	"NK2 transcription factor related, locus 3 (Drosophila) "
7929816	SCD	stearoyl-CoA desaturase (delta-9-desaturase)
7929840	PAX2	paired box 2

7929882	SEMA4G	"sema domain, immunoglobulin domain (Ig), transmembrane domain (TM) and short cytoplasmic domain, (semaphorin) 4G "
7929932	KAZALD1	Kazal-type serine peptidase inhibitor domain 1
7929947	TLX1	T-cell leukemia homeobox 1
7929988	HPS6	Hermansky-Pudlak syndrome 6
7930074	NFKB2	nuclear factor of kappa light polypeptide gene enhancer in B-cells 2 (p49/p100)
7930208	INA	"internexin neuronal intermediate filament protein, alpha "
7930537	TCF7L2	"transcription factor 7-like 2 (T-cell specific, HMG-box) "
7930627	ADRB1	"adrenergic, beta-1-, receptor "
7931181	HMX3	H6 family homeobox 3
7931184	HMX2	H6 family homeobox 2
7931553	UTF1	undifferentiated embryonic cell transcription factor 1
7931810	KLF6	Kruppel-like factor 6
7932186	DCLRE1C	"DNA cross-link repair 1C (PSO2 homolog, S. cerevisiae) "
7932254	ITGA8	"integrin, alpha 8 "
7932390	TRDMT1	tRNA aspartic acid methyltransferase 1
7932938	EPC1	enhancer of polycomb homolog 1 (Drosophila)
7932966	ITGB1	"integrin, beta 1 (fibronectin receptor, beta polypeptide, antigen CD29 includes MDF2, MSK12) "
7932985	NRP1	neuropilin 1
7933194	CXCL12	chemokine (C-X-C motif) ligand 12
7933366	GDF2	growth differentiation factor 2
7933372	GDF10	growth differentiation factor 10
7933469	ARHGAP22	Rho GTPase activating protein 22
7934083	NEUROG3	neurogenin 3
7934215	SPOCK2	"sparc/osteonectin, cwcv and kazal-like domains proteoglycan (testican) 2 "
7934570	KCNMA1	"potassium large conductance calcium-activated channel, subfamily M, alpha member 1 "
7934906	ACTA2	actin, alpha 2, smooth muscle, aorta
7935027	IDE	insulin-degrading enzyme
7935146	NOC3L	nucleolar complex associated 3 homolog (S. cerevisiae)
7935361	SLIT1	slit homolog 1 (Drosophila)
7935528	SFRP5	secreted frizzled-related protein 5
7935707	CHUK	conserved helix-loop-helix ubiquitous kinase
7935776	SCD	stearoyl-CoA desaturase (delta-9-desaturase)
7935855	LBX1	ladybird homeobox 1
7935892	FGF8	fibroblast growth factor 8 (androgen-induced)
7935968	LDB1	LIM domain binding 1
7935990	PSD	pleckstrin and Sec7 domain containing
7936661	PRDX3	peroxiredoxin 3
7936734	FGFR2	fibroblast growth factor receptor 2
7936882	NKX1-2	NK1 homeobox 2
7936904	CTBP2	C-terminal binding protein 2
7937020	MKI67	antigen identified by monoclonal antibody Ki-67
7937079	BNIP3	BCL2/adenovirus E1B 19kDa interacting protein 3
7937335	IFITM1	interferon induced transmembrane protein 1 (9-27)

7937508	CD151	CD151 molecule (Raph blood group)
7937667	BRSK2	BR serine/threonine kinase 2
7937772	IGF2	insulin-like growth factor 2 (somatomedin A)
7938154	ILK	integrin-linked kinase
7938422	CTR9	Ctr9, Paf1/RNA polymerase II complex component, homolog (S. cerevisiae)
7938880	HTATIP2	"HIV-1 Tat interactive protein 2, 30kDa "
7939197	HIPK3	homeodomain interacting protein kinase 3
7939341	CD44	CD44 molecule (Indian blood group)
7939524	EXT2	exostosin 2
7939642	CREB3L1	cAMP responsive element binding protein 3-like 1
7939665	MDK	midkine (neurite growth-promoting factor 2)
7939751	NR1H3	"nuclear receptor subfamily 1, group H, member 3 "
7940530	C11orf9	chromosome 11 open reading frame 9
7940798	MARK2	MAP/microtubule affinity-regulating kinase 2
7940989	ESRRA	estrogen-related receptor alpha
7941457	CCDC85B	coiled-coil domain containing 85B
7941478	SART1	squamous cell carcinoma antigen recognized by T cells
7941917	CABP4	calcium binding protein 4
7942123	CCND1	cyclin D1
7942964	TMEM135	transmembrane protein 135
7943620	ATM	ataxia telangiectasia mutated
7943892	NCAM1	neural cell adhesion molecule 1
7943984	ZBTB16	zinc finger and BTB domain containing 16
7944341	UPK2	uroplakin 2
7944435	HINFP	histone H4 transcription factor
7944493	CBL	Cas-Br-M (murine) ecotropic retroviral transforming sequence
7944537	POU2F3	POU class 2 homeobox 3
7944882	ROBO3	"roundabout, axon guidance receptor, homolog 3 (Drosophila) "
7945014	CHEK1	CHK1 checkpoint homolog (S. pombe)
7945162	BARX2	BARX homeobox 2
7945436	HRAS	v-Ha-ras Harvey rat sarcoma viral oncogene homolog
7945730	ASCL2	achaete-scute complex homolog 2 (Drosophila)
7946610	EIF4G2	eukaryotic translation initiation factor 4 gamma, 2
7946661	DKK3	dickkopf homolog 3 (Xenopus laevis)
7946749	CALCA	calcitonin-related polypeptide alpha
7946757	SOX6	SRY (sex determining region Y)-box 6
7947015	TSG101	tumor susceptibility gene 101
7947199	LGR4	leucine-rich repeat-containing G protein-coupled receptor 4
7947230	BDNF	brain-derived neurotrophic factor
7947363	WT1	Wilms tumor 1
7947540	TRAF6	TNF receptor-associated factor 6
7947652	AMBRA1	autophagy/beclin-1 regulator 1
7947694	CKAP5	cytoskeleton associated protein 5

7947861	SPI1	spleen focus forming virus (SFFV) proviral integration oncogene spi1
7948420	FABP5	fatty acid binding protein 5 (psoriasis-associated)
7948476	PRPF19	PRP19/PSO4 pre-mRNA processing factor 19 homolog (S. cerevisiae)
7948612	FADS1	fatty acid desaturase 1
7949067	BAD	BCL2-associated agonist of cell death
7949206	MEN1	multiple endocrine neoplasia I
7949364	CDCA5	cell division cycle associated 5
7949465	RELA	v-rel reticuloendotheliosis viral oncogene homolog A (avian)
7949532	FOSL1	FOS-like antigen 1
7949754	CLCF1	cardiotrophin-like cytokine factor 1
7950012	CCND1	cyclin D1
7950023	FGF19	fibroblast growth factor 19
7950032	FGF4	fibroblast growth factor 4
7950036	FGF3	fibroblast growth factor 3
7950067	DHCR7	7-dehydrocholesterol reductase
7950136	PHOX2A	paired-like homeobox 2a
7950307	UCP2	uncoupling protein 2 (mitochondrial, proton carrier)
7950321	UCP3	uncoupling protein 3 (mitochondrial, proton carrier)
7950534	WNT11	wingless-type MMTV integration site family, member 11
7950671	GAB2	GRB2-associated binding protein 2
7951046	MRE11A	MRE11 meiotic recombination 11 homolog A (S. cerevisiae)
7951807	CADM1	cell adhesion molecule 1
7952205	MCAM	melanoma cell adhesion molecule
7952268	THY1	Thy-1 cell surface antigen
7952305	BMPR1A	"bone morphogenetic protein receptor, type IA "
7952325	HSPA8	heat shock 70kDa protein 8
7952453	ROBO4	"roundabout homolog 4, magic roundabout (Drosophila) "
7952601	ETS1	v-ets erythroblastosis virus E26 oncogene homolog 1 (avian)
7953012	WNT5B	"wingless-type MMTV integration site family, member 5B "
7953100	FKBP4	"FK506 binding protein 4, 59kDa "
7953135	TULP3	tubby like protein 3
7953200	CCND2	cyclin D2
7953284	NTF3	neurotrophin 3
7953291	CD9	CD9 molecule
7953333	CD27	CD27 molecule
7953547	ATN1	atrophin 1
7953603	C1S	"complement component 1, s subcomponent "
7953651	PEX5	peroxisomal biogenesis factor 5
7953981	ETV6	ets variant 6
7954006	PTMA	prothymosin, alpha
7954029	CDKN1B	cyclin-dependent kinase inhibitor 1B (p27, Kip1)
7954055	APOLD1	apolipoprotein L domain containing 1
7954090	EMP1	epithelial membrane protein 1

7954196	MGST1	microsomal glutathione S-transferase 1
7955063	TMEM106C	transmembrane protein 106C
7955112	H1FNT	"H1 histone family, member N, testis-specific "
7955170	WNT1	"wingless-type MMTV integration site family, member 1 "
7955348	GPD1	glycerol-3-phosphate dehydrogenase 1 (soluble)
7955535	ACVR1B	"activin A receptor, type IB "
7955702	SOAT2	sterol O-acyltransferase 2
7955787	SP1	Sp1 transcription factor
7955890	HNRNPA1	heterogeneous nuclear ribonucleoprotein A1
7956026	GDF11	growth differentiation factor 11
7956038	MMP19	matrix metallopeptidase 19
7956076	CDK2	cyclin-dependent kinase 2
7956120	ERBB3	v-erb-b2 erythroblastic leukemia viral oncogene homolog 3 (avian)
7956287	NAB2	NGFI-A binding protein 2 (EGR1 binding protein 2)
7956910	CAND1	cullin-associated and neddylation-dissociated 1
7958051	ASCL1	achaete-scute complex homolog 1 (Drosophila)
7958620	IFT81	intraflagellar transport 81 homolog (Chlamydomonas)
7958749	SH2B3	SH2B adaptor protein 3
7958931	DTX1	deltex homolog 1 (Drosophila)
7959786	AACS	acetoacetyl-CoA synthetase
7959957	ULK1	unc-51-like kinase 1 (C. elegans)
7960340	FOXM1	forkhead box M1
7960397	FGF23	fibroblast growth factor 23
7960518	TNFRSF1A	tumor necrosis factor receptor superfamily, member 1A
7960850	SLC2A14	"solute carrier family 2 (facilitated glucose transporter), member 14 "
7960865	SLC2A3	solute carrier family 2 (facilitated glucose transporter), member 3
7960933	M6PR	mannose-6-phosphate receptor (cation dependent)
7961022	PTMA	prothymosin, alpha
7961798	SOX5	SRY (sex determining region Y)-box 5
7961865	KRAS	v-Ki-ras2 Kirsten rat sarcoma viral oncogene homolog
7961891	BHLHE41	"basic helix-loop-helix family, member e41 "
7962000	PTHLH	parathyroid hormone-like hormone
7962579	AMIGO2	adhesion molecule with Ig-like domain 2
7962659	HDAC7	histone deacetylase 7
7962689	VDR	vitamin D (1,25- dihydroxyvitamin D3) receptor
7962703	COL2A1	"collagen, type II, alpha 1 "
7962831	CCNT1	cyclin T1
7962918	WNT10B	"wingless-type MMTV integration site family, member 10B "
7963020	DHH	desert hedgehog homolog (Drosophila)
7963157	RACGAP1	Rac GTPase activating protein 1
7963375	KRT84	keratin 84
7963421	KRT6A	keratin 6A
7963523	KRT3	keratin 3

7963534	KRT4	keratin 4
7963664	SP7	Sp7 transcription factor
7963880	ITGA7	integrin, alpha 7
7963946	MMP19	matrix metallopeptidase 19
7964522	CDK4	cyclin-dependent kinase 4
7965090	CSRP2	cysteine and glycine-rich protein 2
7965322	KITLG	KIT ligand
7965335	DUSP6	dual specificity phosphatase 6
7965359	ATP2B1	ATPase, Ca++ transporting, plasma membrane 1
7965423	BTG1	"B-cell translocation gene 1, anti-proliferative "
7965812	GNPTAB	"N-acetylglucosamine-1-phosphate transferase, alpha and beta subunits "
7966189	FOXN4	forkhead box N4
7966631	LHX5	LIM homeobox 5
7966668	TBX5	T-box 5
7966690	TBX3	T-box 3
7966749	TESC	tescalcin
7966878	CIT	"citron (rho-interacting, serine/threonine kinase 21) "
7967039	MSI1	musashi homolog 1 (Drosophila)
7967175	KDM2B	lysine (K)-specific demethylase 2B
7967230	DIABLO	diablo homolog (Drosophila)
7967685	STX2	syntaxin 2
7968015	TNFRSF19	tumor necrosis factor receptor superfamily, member 19
7968199	CDK8	cyclin-dependent kinase 8
7968260	GSX1	GS homeobox 1
7968265	PDX1	pancreatic and duodenal homeobox 1
7968274	PAN3	PAN3 poly(A) specific ribonuclease subunit homolog (S. cerevisiae)
7969017	RB1	retinoblastoma 1
7969271	SUGT1	SGT1, suppressor of G2 allele of SKP1 (S. cerevisiae)
7969830	ZIC2	"Zic family member 2 (odd-paired homolog, Drosophila) "
7970317	TFDP1	transcription factor Dp-1
7970347	CDC16	cell division cycle 16 homolog (S. cerevisiae)
7970727	CDX2	caudal type homeobox 2
7970763	FLT1	fms-related tyrosine kinase 1 (vascular endothelial growth factor/vascular permeability factor receptor)
7970954	DCLK1	doublecortin-like kinase 1
7971015	SMAD9	SMAD family member 9
7971177	FOXO1	forkhead box O1
7972069	MYCBP2	MYC binding protein 2
7972157	EDNRB	endothelin receptor type B
7972217	SPRY2	sprouty homolog 2 (Drosophila)
7972336	DZIP1	DAZ interacting protein 1
7972567	ZIC5	"Zic family member 5 (odd-paired homolog, Drosophila) "
7972713	EFNB2	ephrin-B2
7972737	LIG4	"ligase IV, DNA, ATP-dependent "

7973067	PNP	purine nucleoside phosphorylase
7973084	ANG	"angiogenin, ribonuclease, RNase A family, 5 "
7973336	MMP14	matrix metallopeptidase 14 (membrane-inserted)
7973371	ACIN1	apoptotic chromatin condensation inducer 1
7973530	PCK2	phosphoenolpyruvate carboxykinase 2 (mitochondrial)
7973709	NFATC4	"nuclear factor of activated T-cells, cytoplasmic, calcineurin-dependent 4 "
7973743	BNIP3	BCL2/adenovirus E1B 19kDa interacting protein 3
7973745	FOXG1	forkhead box G1
7973974	PAX9	paired box 9
7974404	CDKN3	cyclin-dependent kinase inhibitor 3
7974461	LGALS3	"lectin, galactoside-binding, soluble, 3 "
7974799	MNAT1	menage a trois homolog 1, cyclin H assembly factor (Xenopus laevis)
7974851	HIF1A	"hypoxia inducible factor 1, alpha subunit (basic helix-loop-helix transcription factor) "
7975545	PSEN1	presenilin 1
7975595	C14orf169	chromosome 14 open reading frame 169
7975602	ACOT2	acyl-CoA thioesterase 2
7975696	VSX2	visual system homeobox 2
7975779	FOS	FBJ murine osteosarcoma viral oncogene homolog
7976012	NRXN3	neurexin 3
7976128	TTC8	tetratricopeptide repeat domain 8
7976567	BDKRB1	bradykinin receptor B1
7976783	DLK1	delta-like 1 homolog (Drosophila)
7976863	PPP2R5C	protein phosphatase 2, regulatory subunit B', gamma
7977046	TNFAIP2	"tumor necrosis factor, alpha-induced protein 2 "
7977511	TEP1	telomerase-associated protein 1
7977621	NDRG2	NDRG family member 2
7977657	HNRNPC	heterogeneous nuclear ribonucleoprotein C (C1/C2)
7977906	ACIN1	apoptotic chromatin condensation inducer 1
7977928	CEBPE	"CCAAT/enhancer binding protein (C/EBP), epsilon "
7978222	TGM1	"transglutaminase 1 (K polypeptide epidermal type I, protein-glutamine-gamma-glutamyltransferase) "
7978407	PRKD1	protein kinase D1
7978644	NFKBIA	"nuclear factor of kappa light polypeptide gene enhancer in B-cells inhibitor, alpha "
7978706	FOXA1	forkhead box A1
7979179	ERO1L	ERO1-like (S. cerevisiae)
7979241	BMP4	bone morphogenetic protein 4
7979307	DLGAP5	discs, large (Drosophila) homolog-associated protein 5
7979357	OTX2	orthodenticle homeobox 2
7979505	SIX1	SIX homeobox 1
7979510	SIX4	SIX homeobox 4
7979824	ACTN1	actinin, alpha 1
7980005	NUMB	numb homolog (Drosophila)
7980098	ALDH6A1	"aldehyde dehydrogenase 6 family, member A1 "
7980233	PGF	placental growth factor

7980316	TGFB3	"transforming growth factor, beta 3 "
7981111	DICER1	"dicer 1, ribonuclease type III "
7981242	BCL11B	B-cell CLL/lymphoma 11B (zinc finger protein)
7981494	AKT1	v-akt murine thymoma viral oncogene homolog 1
7981538	JAG2	jagged 2
7981824	CYFIP1	cytoplasmic FMR1 interacting protein 1
7981917	C15orf2	chromosome 15 open reading frame 2
7982204	HMGN2	high-mobility group nucleosomal binding domain 2
7982597	THBS1	thrombospondin 1
7982792	RAD51	RAD51 homolog (RecA homolog, E. coli) (S. cerevisiae)
7982854	DLL4	delta-like 4 (Drosophila)
7983502	PLDN	pallidin homolog (mouse)
7983527	SEMA6D	"sema domain, transmembrane domain (TM), and cytoplasmic domain, (semaphorin) 6D "
7983606	EID1	EP300 interacting inhibitor of differentiation 1
7983630	FGF7	fibroblast growth factor 7
7983969	CCNB2	cyclin B2
7984319	MAP2K1	mitogen-activated protein kinase kinase 1
7984364	SMAD3	SMAD family member 3
7984524	PAQR5	progestin and adipoQ receptor family member V
7984686	BBS4	Bardet-Biedl syndrome 4
7984743	CD276	CD276 molecule
7984779	PML	promyelocytic leukemia
7985080	ISL2	ISL LIM homeobox 2
7985577	ZSCAN2	zinc finger and SCAN domain containing 2
7985786	ACAN	aggrecan
7985934	SEMA4B	"sema domain, immunoglobulin domain (Ig), transmembrane domain (TM) and short cytoplasmic domain, (semaphorin) 4B "
7985983	NGRN	"neugrin, neurite outgrowth associated "
7986068	BLM	"Bloom syndrome, RecQ helicase-like "
7986160	UNC45A	unc-45 homolog A (C. elegans)
7986359	IGF1R	insulin-like growth factor 1 receptor
7986383	IGF1R	insulin-like growth factor 1 receptor
7986411	MEF2A	myocyte enhancer factor 2A
7987315	ACTC1	actin, alpha, cardiac muscle 1
7987405	RASGRP1	RAS guanyl releasing protein 1 (calcium and DAG-regulated)
7987536	FAM82A2	"family with sequence similarity 82, member A2 "
7988031	RPS3A	ribosomal protein S3A
7988987	ONECUT1	one cut homeobox 1
7989023	RAB27A	"RAB27A, member RAS oncogene family "
7989365	RORA	RAR-related orphan receptor A
7989985	ITGA11	integrin, alpha 11
7990345	SEMA7A	"semaphorin 7A, GPI membrane anchor (John Milton Hagen blood group) "
7990545	CSPG4	chondroitin sulfate proteoglycan 4
7990736	ADAMTS7	ADAM metallopeptidase with thrombospondin type 1 motif, 7

7991313	PLIN1	perilipin 1
7991323	PEX11A	peroxisomal biogenesis factor 11 alpha
7991332	MESP1	mesoderm posterior 1 homolog (mouse)
7991335	ANPEP	alanyl (membrane) aminopeptidase
7992038	METRN	"meteorin, glial cell differentiation regulator "
7992409	RNF151	ring finger protein 151
7992594	CCNF	cyclin F
7992789	TNFRSF12A	"tumor necrosis factor receptor superfamily, member 12A "
7992956	GLIS2	GLIS family zinc finger 2
7992973	DNAJA3	"DnaJ (Hsp40) homolog, subfamily A, member 3 "
7993310	MKL2	MKL/myocardin-like 2
7993467	NDE1	nudE nuclear distribution gene E homolog 1 (A. nidulans)
7994280	IL4R	interleukin 4 receptor
7994487	CD19	CD19 molecule
7994939	RNF40	ring finger protein 40
7995055	MYST1	MYST histone acetyltransferase 1
7995206	TGFB1I1	transforming growth factor beta 1 induced transcript 1
7995574	HNRNPA1	heterogeneous nuclear ribonucleoprotein A1
7995631	RBL2	retinoblastoma-like 2 (p130)
7995668	IRX5	iroquois homeobox 5
7995681	MMP2	"matrix metallopeptidase 2 (gelatinase A, 72kDa gelatinase, 72kDa type IV collagenase) "
7996219	NDRG4	NDRG family member 4
7996430	HSF4	heat shock transcription factor 4
7996455	E2F4	E2F transcription factor 4, p107/p130-binding
7996647	PARD6A	par-6 partitioning defective 6 homolog alpha (C. elegans)
7996785	PRMT7	protein arginine methyltransferase 7
7997352	WWOX	WW domain containing oxidoreductase
7997533	OSGIN1	oxidative stress induced growth inhibitor 1
7997726	FOXF1	forkhead box F1
7997733	FOXC2	"forkhead box C2 (MFH-1, mesenchyme forkhead 1) "
7997801	IL17C	interleukin 17C
7998055	MC1R	melanocortin 1 receptor (alpha melanocyte stimulating hormone receptor)
7998063	TUBB3	tubulin, beta 3
7999044	CREBBP	CREB binding protein
7999423	SOCS1	suppressor of cytokine signaling 1
7999431	PRM3	protamine 3
8000409	HMGN2	high-mobility group nucleosomal binding domain 2
8000567	IL27	interleukin 27
8001306	SIAH1	seven in absentia homolog 1 (Drosophila)
8001387	SALL1	sal-like 1 (Drosophila)
8001449	IRX3	iroquois homeobox 3
8001507	BBS2	Bardet-Biedl syndrome 2
8001800	CDH11	"cadherin 11, type 2, OB-cadherin (osteoblast) "

8003068	MPHOSPH6	M-phase phosphoprotein 6
8003204	GINS2	GINS complex subunit 2 (Psf2 homolog)
8003298	SLC7A5	"solute carrier family 7 (cationic amino acid transporter, y+ system), member 5 "
8003425	CBFA2T3	"core-binding factor, runt domain, alpha subunit 2; translocated to, 3 "
8003758	PAFAH1B1	"platelet-activating factor acetylhydrolase 1b, regulatory subunit 1 (45kDa) "
8004309	SLC2A4	"solute carrier family 2 (facilitated glucose transporter), member 4 "
8004360	KCTD11	potassium channel tetramerisation domain containing 11
8004394	SPEM1	spermatid maturation 1
8004408	FGF11	fibroblast growth factor 11
8004464	TNFSF12	tumor necrosis factor (ligand) superfamily, member 12
8004571	EFNB3	ephrin-B3
8004784	ALOX15B	"arachidonate 15-lipoxygenase, type B "
8004867	NDEL1	nudE nuclear distribution gene E homolog (A. nidulans)-like 1
8005048	MYOCD	myocardin
8005576	MAPK7	mitogen-activated protein kinase 7
8006239	NF1	neurofibromin 1
8006409	CDK5R1	"cyclin-dependent kinase 5, regulatory subunit 1 (p35) "
8006531	SLFN5	schlafen family member 5
8006638	GGNBP2	gametogenetin binding protein 2
8006681	LHX1	LIM homeobox 1
8006768	SOCS7	suppressor of cytokine signaling 7
8006906	ERBB2	"v-erb-b2 erythroblastic leukemia viral oncogene homolog 2, neuro/glioblastoma derived oncogene homolog (avian) "
8006940	GRB7	growth factor receptor-bound protein 7
8006999	CSF3	colony stimulating factor 3 (granulocyte)
8007008	THRA	"thyroid hormone receptor, alpha (erythroblastic leukemia viral (v-erb-a) oncogene homolog, avian) "
8007071	CDC6	cell division cycle 6 homolog (S. cerevisiae)
8007084	RARA	"retinoic acid receptor, alpha "
8007212	STAT5A	signal transducer and activator of transcription 5A
8007637	FZD2	frizzled homolog 2 (Drosophila)
8007745	HEXIM1	hexamethylene bis-acetamide inducible 1
8007895	WNT9B	wingless-type MMTV integration site family, member 9B
8007931	ITGB3	"integrin, beta 3 (platelet glycoprotein IIIa, antigen CD61) "
8008074	CDK5RAP3	CDK5 regulatory subunit associated protein 3
8008151	IGF2BP1	insulin-like growth factor 2 mRNA binding protein 1
8008201	NGFR	nerve growth factor receptor
8008388	SPATA20	spermatogenesis associated 20
8008517	NME1	"non-metastatic cells 1, protein (NM23A) expressed in "
8008627	NOG	noggin
8008682	MSI2	musashi homolog 2 (Drosophila)
8009096	ACE	angiotensin I converting enzyme (peptidyl-dipeptidase A) 1
8009227	PSMC5	proteasome (prosome, macropain) 26S subunit, ATPase, 5
8009301	PRKCA	"protein kinase C, alpha "
8009417	KPNA2	karyopherin alpha 2 (RAG cohort 1, importin alpha 1)

8009517	SOX9	SRY (sex determining region Y)-box 9
8010967	NXN	nucleoredoxin
8012257	TP53	tumor protein p53
8012403	AURKB	aurora kinase B
8012605	GAS7	growth arrest-specific 7
8012896	PMP22	peripheral myelin protein 22
8012953	TRIM16	tripartite motif-containing 16
8013135	SREBF1	sterol regulatory element binding transcription factor 1
8013788	FLOT2	flotillin 2
8014214	NLE1	notchless homolog 1 (Drosophila)
8014841	MED1	mediator complex subunit 1
8014865	NEUROD2	neurogenic differentiation 2
8014974	TOP2A	topoisomerase (DNA) II alpha 170kDa
8015366	KRT14	keratin 14
8015590	STAT5B	signal transducer and activator of transcription 5B
8015607	STAT3	signal transducer and activator of transcription 3 (acute-phase response factor)
8015769	BRCA1	breast cancer 1, early onset
8015827	SOST	sclerostin
8015914	HDAC5	histone deacetylase 5
8016311	WNT3	wingless-type MMTV integration site family, member 3
8016433	HOXB1	homeobox B1
8016452	HOXB4	homeobox B4
8016578	SLC35B1	solute carrier family 35, member B1
8016628	PPP1R9B	"protein phosphatase 1, regulatory (inhibitor) subunit 9B "
8016640	HILS1	"histone linker H1 domain, spermatid-specific 1 "
8016646	COL1A1	"collagen, type I, alpha 1 "
8016739	TOB1	"transducer of ERBB2, 1 "
8016745	SPAG9	sperm associated antigen 9
8017150	TUBD1	"tubulin, delta 1 "
8017711	GNA13	"guanine nucleotide binding protein (G protein), alpha 13 "
8017718	AXIN2	axin 2
8018258	USH1G	Usher syndrome 1G (autosomal recessive)
8018264	C17orf28	chromosome 17 open reading frame 28
8018364	GRB2	growth factor receptor-bound protein 2
8018579	EVPL	envoplakin
8018646	FOXJ1	forkhead box J1
8018793	JMJD6	jumonji domain containing 6
8018864	SOCS3	suppressor of cytokine signaling 3
8018966	TIMP2	TIMP metallopeptidase inhibitor 2
8018972	TIMP2	TIMP metallopeptidase inhibitor 2
8019108	AZI1	5-azacytidine induced 1
8019308	MAFG	v-maf musculoaponeurotic fibrosarcoma oncogene homolog G (avian)
8019367	RFNG	RFNG O-fucosylpeptide 3-beta-N-acetylglucosaminyltransferase

8019392	FASN	fatty acid synthase
8019737	KPNA2	karyopherin alpha 2 (RAG cohort 1, importin alpha 1)
8019796	MAFG	v-maf musculoaponeurotic fibrosarcoma oncogene homolog G (avian)
8019798	MAFG	v-maf musculoaponeurotic fibrosarcoma oncogene homolog G (avian)
8020090	TWSG1	twisted gastrulation homolog 1 (Drosophila)
8020423	MIB1	mindbomb homolog 1 (Drosophila)
8020455	GATA6	GATA binding protein 6
8020468	RBBP8	retinoblastoma binding protein 8
8021365	ATP8B1	ATPase, aminophospholipid transporter, class I, type 8B, member 1
8021418	MALT1	mucosa associated lymphoid tissue lymphoma translocation gene 1
8021866	NFATC1	nuclear factor of activated T-cells, cytoplasmic, calcineurin-dependent 1
8022424	NF1	neurofibromin 1
8022814	HNRNPA1	heterogeneous nuclear ribonucleoprotein A1
8023497	ATP8B1	ATPase, aminophospholipid transporter, class I, type 8B, member 1
8023646	BCL2	B-cell CLL/lymphoma 2
8023941	MADCAM1	mucosal vascular addressin cell adhesion molecule 1
8023948	C19orf20	chromosome 19 open reading frame 20
8023968	CDC34	cell division cycle 34 homolog (S. cerevisiae)
8023990	FGF22	fibroblast growth factor 22
8023995	FSTL3	follistatin-like 3 (secreted glycoprotein)
8024062	CFD	complement factor D (adipsin)
8024282	DAZAP1	DAZ associated protein 1
8024429	AMH	anti-Mullerian hormone
8024485	GADD45B	"growth arrest and DNA-damage-inducible, beta "
8024557	GNA11	"guanine nucleotide binding protein (G protein), alpha 11 (Gq class) "
8024566	GNA11	"guanine nucleotide binding protein (G protein), alpha 11 (Gq class) "
8024728	ITGB1BP3	integrin beta 1 binding protein 3
8024754	CREB3L3	cAMP responsive element binding protein 3-like 3
8025179	ZNF358	zinc finger protein 358
8025278	RETN	resistin
8025402	ANGPTL4	angiopoietin-like 4
8025601	ICAM1	intercellular adhesion molecule 1
8026047	JUNB	jun B proto-oncogene
8026106	CALR	calreticulin
8026214	NANOS3	nanos homolog 3 (Drosophila)
8027402	CCNE1	cyclin E1
8027566	CEBPG	"CCAAT/enhancer binding protein (C/EBP), gamma "
8027778	FXYD5	FXYD domain containing ion transport regulator 5
8028162	TBCB	tubulin folding cofactor B
8028652	ZFP36	"zinc finger protein 36, C3H type, homolog (mouse) "
8028719	DLL3	delta-like 3 (Drosophila)
8028872	LTBP4	latent transforming growth factor beta binding protein 4
8029006	AXL	AXL receptor tyrosine kinase

8029129	RPS19	ribosomal protein S19
8029465	BCL3	B-cell CLL/lymphoma 3
8029530	APOE	apolipoprotein E
8029560	CLPTM1	cleft lip and palate associated transmembrane protein 1
8029580	RELB	v-rel reticuloendotheliosis viral oncogene homolog B
8029693	FOSB	FBJ murine osteosarcoma viral oncogene homolog B
8029754	FOXA3	forkhead box A3
8030339	FLT3LG	fms-related tyrosine kinase 3 ligand
8030557	ATF5	activating transcription factor 5
8030630	NR1H2	"nuclear receptor subfamily 1, group H, member 2 "
8030881	PPP2R1A	"protein phosphatase 2, regulatory subunit A, alpha "
8031441	BRSK1	BR serine/threonine kinase 1
8032023	THEG	Theg homolog (mouse)
8032290	TCF3	transcription factor 3 (E2A immunoglobulin enhancer binding factors E12/E47)
8032392	MKNK2	MAP kinase interacting serine/threonine kinase 2
8032718	DAPK3	death-associated protein kinase 3
8032755	ZBTB7A	zinc finger and BTB domain containing 7A
8032834	LRG1	leucine-rich alpha-2-glycoprotein 1
8032839	SEMA6B	"sema domain, transmembrane domain (TM), and cytoplasmic domain, (semaphorin) 6B "
8033362	INSR	insulin receptor
8033892	ANGPTL6	angiopoietin-like 6
8033912	DNMT1	DNA (cytosine-5-)-methyltransferase 1
8034034	KEAP1	kelch-like ECH-associated protein 1
8034043	S1PR5	sphingosine-1-phosphate receptor 5
8034544	PRDX2	peroxiredoxin 2
8034565	DNASE2	"deoxyribonuclease II, lysosomal "
8034643	CACNA1A	"calcium channel, voltage-dependent, P/Q type, alpha 1A subunit "
8034772	ASF1B	ASF1 anti-silencing function 1 homolog B (S. cerevisiae)
8034940	NOTCH3	Notch homolog 3 (Drosophila)
8035445	JUND	jun D proto-oncogene
8035847	ZNF675	zinc finger protein 675
8036004	CEBPA	"CCAAT/enhancer binding protein (C/EBP), alpha "
8036133	UPK1A	uroplakin 1A
8036494	GGN	gametogenetin
8036749	EID2	EP300 interacting inhibitor of differentiation 2
8037005	TGFB1	"transforming growth factor, beta 1 "
8037186	LIPE	lipase, hormone-sensitive
8037537	ERCC2	"excision repair cross-complementing rodent repair deficiency, complementation group 2 "
8037732	NANOS2	nanos homolog 2 (Drosophila)
8037775	PTGIR	prostaglandin I2 (prostacyclin) receptor (IP)
8037913	NAPA	"N-ethylmaleimide-sensitive factor attachment protein, alpha "
8037931	ZNF541	zinc finger protein 541
8039484	IL11	interleukin 11

8040070	SOX11	SRY (sex determining region Y)-box 11
8040103	ID2	"inhibitor of DNA binding 2, dominant negative helix-loop-helix protein "
8040142	CPSF3	cleavage and polyadenylation specific factor 3, 73kDa
8040223	RRM2	ribonucleotide reductase M2
8040456	MSGN1	mesogenin 1
8040473	RHOB	"ras homolog gene family, member B "
8040479	GDF7	growth differentiation factor 7
8040578	CENPO	centromere protein O
8040827	TCF23	transcription factor 23
8040898	TRIM54	tripartite motif-containing 54
8041236	SPAST	spastin
8041447	CRIM1	cysteine rich transmembrane BMP regulator 1 (chordin-like)
8041582	PKDCC	"protein kinase domain containing, cytoplasmic homolog (mouse) "
8041781	EPAS1	endothelial PAS domain protein 1
8041820	SOCS5	suppressor of cytokine signaling 5
8041867	MSH2	"mutS homolog 2, colon cancer, nonpolyposis type 1 (E. coli) "
8042439	ANTXR1	anthrax toxin receptor 1
8042487	GMCL1	germ cell-less homolog 1 (Drosophila)
8042537	ATP6V1B1	"ATPase, H+ transporting, lysosomal 56/58kDa, V1 subunit B1 "
8042701	EMX1	empty spiracles homeobox 1
8042737	ALMS1	Alstrom syndrome 1
8042925	SEMA4F	"sema domain, immunoglobulin domain (Ig), transmembrane domain (TM) and short cytoplasmic domain, (semaphorin) 4F "
8043114	TCF7L1	"transcription factor 7-like 1 (T-cell specific, HMG-box) "
8043203	VAMP5	vesicle-associated membrane protein 5 (myobrevin)
8043244	ATOH8	atonal homolog 8 (Drosophila)
8043283	KDM3A	lysine (K)-specific demethylase 3A
8043504	MAL	"mal, T-cell differentiation protein "
8044686	ACTR3	ARP3 actin-related protein 3 homolog (yeast)
8044927	INHBB	"inhibin, beta B "
8044933	GLI2	GLI family zinc finger 2
8045381	CCNT2	cyclin T2
8045587	ACVR2A	"activin A receptor, type IIA "
8045816	GPD2	glycerol-3-phosphate dehydrogenase 2 (mitochondrial)
8046373	DLX1	distal-less homeobox 1
8046380	ITGA6	"integrin, alpha 6 "
8046461	ZAK	sterile alpha motif and leucine zipper containing kinase AZK
8046628	RBM45	RNA binding motif protein 45
8046646	OSBPL6	oxysterol binding protein-like 6
8046695	ITGA4	"integrin, alpha 4 (antigen CD49D, alpha 4 subunit of VLA-4 receptor) "
8046861	ITGAV	"integrin, alpha V (vitronectin receptor, alpha polypeptide, antigen CD51) "
8046922	COL3A1	collagen, type III, alpha 1
8047059	HNRNPC	heterogeneous nuclear ribonucleoprotein C (C1/C2)
8047086	NAB1	NGFI-A binding protein 1 (EGR1 binding protein 1)

8047174	SLC39A10	solute carrier family 39 (zinc transporter), member 10
8047419	CASP8	"caspase 8, apoptosis-related cysteine peptidase "
8047487	FZD7	frizzled homolog 7 (Drosophila)
8047538	BMPR2	"bone morphogenetic protein receptor, type II (serine/threonine kinase) "
8047738	NRP2	neuropilin 2
8047839	CREB1	cAMP responsive element binding protein 1
8048146	XRCC5	X-ray repair complementing defective repair in Chinese hamster cells 5 (double-strand-break rejoining)
8048340	RQCD1	RCD1 required for cell differentiation1 homolog (S. pombe)
8048445	WNT6	wingless-type MMTV integration site family, member 6
8048452	WNT10A	wingless-type MMTV integration site family, member 10A
8048541	DES	desmin
8048551	SPEG	SPEG complex locus
8048847	AGFG1	ArfGAP with FG repeats 1
8049083	PTMA	prothymosin, alpha
8049487	MLPH	melanophilin
8050190	ADAM17	ADAM metallopeptidase domain 17
8050215	YWHAQ	tyrosine 3-monooxygenase/tryptophan 5-monooxygenase activation protein, theta polypeptide
8050497	OSR1	odd-skipped related 1 (Drosophila)
8050763	CENPO	centromere protein O
8051762	SLC8A1	solute carrier family 8 (sodium/calcium exchanger), member 1
8051949	SIX2	SIX homeobox 2
8051993	PIGF	phosphatidylinositol glycan anchor biosynthesis, class F
8052149	PSME4	"proteasome (prosome, macropain) activator subunit 4 "
8052399	BCL11A	B-cell CLL/lymphoma 11A (zinc finger protein)
8052762	GFPT1	glutamine--fructose-6-phosphate transaminase 1
8053263	SEMA4F	"sema domain, immunoglobulin domain (Ig), transmembrane domain (TM) and short cytoplasmic domain, (semaphorin) 4F "
8053668	EIF2AK3	eukaryotic translation initiation factor 2-alpha kinase 3
8054004	SEMA4C	"sema domain, immunoglobulin domain (Ig), transmembrane domain (TM) and short cytoplasmic domain, (semaphorin) 4C "
8054377	FHL2	four and a half LIM domains 2
8054664	ZC3H8	zinc finger CCCH-type containing 8
8054740	PAX8	paired box 8
8054945	BIN1	bridging integrator 1
8054978	ERCC3	"excision repair cross-complementing rodent repair deficiency, complementation group 3 (xeroderma pigmentosum group B complementing) "
8055952	NR4A2	"nuclear receptor subfamily 4, group A, member 2 "
8055992	ACVR1C	"activin A receptor, type IC "
8056005	ACVR1	"activin A receptor, type I "
8056222	DPP4	dipeptidyl-peptidase 4
8056257	FAP	fibroblast activation protein, alpha
8056784	DLX2	distal-less homeobox 2
8056798	SP3	Sp3 transcription factor
8056909	ATF2	activating transcription factor 2
8056943	KIAA1715	KIAA1715
8057506	FRZB	frizzled-related protein

8057620	COL5A2	"collagen, type V, alpha 2 "
8058857	IGFBP5	insulin-like growth factor binding protein 5
8058914	AAMP	"angio-associated, migratory cell protein "
8059014	FEV	FEV (ETS oncogene family)
8059067	IHH	Indian hedgehog homolog (Drosophila)
8059071	NHEJ1	nonhomologous end-joining factor 1
8059376	SERPINE2	"serpin peptidase inhibitor, clade E (nexin, plasminogen activator inhibitor type 1), member 2 "
8059525	TM4SF20	transmembrane 4 L six family member 20
8059580	DNER	delta/notch-like EGF repeat containing
8059739	NPPC	natriuretic peptide precursor C
8059783	NGEF	neuronal guanine nucleotide exchange factor
8059989	HES6	hairy and enhancer of split 6 (Drosophila)
8060030	HDAC4	histone deacetylase 4
8060286	DTYMK	deoxythymidylate kinase (thymidylate kinase)
8060334	SOX12	SRY (sex determining region Y)-box 12
8060604	OXT	"oxytocin, prepropeptide "
8060850	BMP2	bone morphogenetic protein 2
8061136	PTMA	prothymosin, alpha
8061303	INSM1	insulinoma-associated 1
8061357	PAX1	paired box 1
8061564	ID1	inhibitor of DNA binding 1, dominant negative helix-loop-helix protein
8061746	DNMT3B	DNA (cytosine-5-)-methyltransferase 3 beta
8062134	GDF5	growth differentiation factor 5
8062319	TGIF2	TGFB-induced factor homeobox 2
8062395	NNAT	neuronatin
8062527	ADIG	adipogenin
8062603	TOP1	topoisomerase (DNA) I
8062705	L3MBTL	l(3)mbt-like (Drosophila)
8062981	PIGT	"phosphatidylinositol glycan anchor biosynthesis, class T "
8063043	UBE2C	ubiquitin-conjugating enzyme E2C
8063115	MMP9	"matrix metallopeptidase 9 (gelatinase B, 92kDa gelatinase, 92kDa type IV collagenase) "
8063369	RNF114	ring finger protein 114
8063386	CEBPB	"CCAAT/enhancer binding protein (C/EBP), beta "
8063536	TFAP2C	transcription factor AP-2 gamma (activating enhancer binding protein 2 gamma)
8063668	GNAS	GNAS complex locus
8064100	PPDPF	pancreatic progenitor cell differentiation and proliferation factor homolog (zebrafish)
8064277	MYT1	myelin transcription factor 1
8064844	PCNA	proliferating cell nuclear antigen
8064967	MKKS	McKusick-Kaufman syndrome
8064978	JAG1	jagged 1 (Alagille syndrome)
8065334	NKX2-2	NK2 homeobox 2
8065344	FOXA2	forkhead box A2
8065668	CDK5RAP1	CDK5 regulatory subunit associated protein 1

8065776	NCOA6	nuclear receptor coactivator 6
8065905	GDF5	growth differentiation factor 5
8066051	NDRG3	NDRG family member 3
8066136	RBL1	retinoblastoma-like 1 (p107)
8066431	ADA	adenosine deaminase
8066619	PLTP	phospholipid transfer protein
8066953	SPATA2	spermatogenesis associated 2
8067055	ATP9A	ATPase, class II, type 9A
8067409	LAMA5	"laminin, alpha 5 "
8067521	GATA5	GATA binding protein 5
8067554	TCFL5	transcription factor-like 5 (basic helix-loop-helix)
8067862	NF1	neurofibromin 1
8068024	JAM2	junctional adhesion molecule 2
8068168	SOD1	"superoxide dismutase 1, soluble "
8068496	SIM2	single-minded homolog 2 (Drosophila)
8068522	TTC3	tetratricopeptide repeat domain 3
8068866	PKNOX1	PBX/knotted 1 homeobox 1
8069269	COL6A1	collagen, type VI, alpha 1
8069301	COL6A2	collagen, type VI, alpha 2
8070182	RCAN1	regulator of calcineurin 1
8070194	RUNX1	runt-related transcription factor 1
8070665	SIK1	salt-inducible kinase 1
8071768	SMARCB1	SWI/SNF related, matrix associated, actin dependent regulator of chromatin, subfamily b, member 1
8072577	YWHAH	"tyrosine 3-monooxygenase/tryptophan 5-monooxygenase activation protein, eta polypeptide "
8072687	MCM5	minichromosome maintenance complex component 5
8072876	LGALS1	"lectin, galactoside-binding, soluble, 1 "
8073007	MAFF	v-maf musculoaponeurotic fibrosarcoma oncogene homolog F (avian)
8073022	CBY1	chibby homolog 1 (Drosophila)
8073148	ATF4	activating transcription factor 4 (tax-responsive enhancer element B67)
8073522	SREBF2	sterol regulatory element binding transcription factor 2
8073733	NUP50	nucleoporin 50kDa
8073743	UPK3A	uroplakin 3A
8073826	PPARA	peroxisome proliferator-activated receptor alpha
8074791	MAPK1	mitogen-activated protein kinase 1
8075239	THOC5	THO complex 5
8075310	LIF	leukemia inhibitory factor (cholinergic differentiation factor)
8075493	PATZ1	POZ (BTB) and AT hook containing zinc finger 1
8075728	MYH9	"myosin, heavy chain 9, non-muscle "
8075992	SOX10	SRY (sex determining region Y)-box 10
8076185	CBX7	chromobox homolog 7
8076331	TOB2	"transducer of ERBB2, 2 "
8076441	NFAM1	NFAT activating protein with ITAM motif 1
8076586	SCUBE1	"signal peptide, CUB domain, EGF-like 1 "

8076734	WNT7B	wingless-type MMTV integration site family, member 7B
8076894	MLC1	megalencephalic leukoencephalopathy with subcortical cysts 1
8076962	MAPK12	mitogen-activated protein kinase 12
8077103	TYMP	thymidine phosphorylase
8077262	DTYMK	deoxythymidylate kinase (thymidylate kinase)
8077503	CAV3	caveolin 3
8077781	VHL	von Hippel-Lindau tumor suppressor
8077899	PPARG	peroxisome proliferator-activated receptor gamma
8078286	RARB	"retinoic acid receptor, beta "
8078350	TGFBR2	"transforming growth factor, beta receptor II (70/80kDa) "
8078600	TCEA1	"transcription elongation factor A (SII), 1 "
8078898	SLC25A38	"solute carrier family 25, member 38 "
8079021	CTNNB1	"catenin (cadherin-associated protein), beta 1, 88kDa "
8079140	SNRK	SNF related kinase
8079153	ABHD5	abhydrolase domain containing 5
8080714	FLNB	"filamin B, beta "
8080938	MITF	microphthalmia-associated transcription factor
8081001	ROBO2	"roundabout, axon guidance receptor, homolog 2 (Drosophila) "
8081235	COL8A1	collagen, type VIII, alpha 1
8081657	CD200	CD200 molecule
8081686	BOC	Boc homolog (mouse)
8081740	ATP6V1A	ATPase, H+ transporting, lysosomal 70kDa, V1 subunit A
8081810	GAP43	growth associated protein 43
8082350	MCM2	minichromosome maintenance complex component 2
8083240	AGTR1	"angiotensin II receptor, type 1 "
8083429	MBNL1	muscleblind-like (Drosophila)
8083494	MME	membrane metallo-endopeptidase
8083594	PTX3	pentraxin 3, long
8083616	MLF1	myeloid leukemia factor 1
8083887	CLDN11	claudin 11
8083901	FNDC3B	fibronectin type III domain containing 3B
8083968	NLGN1	neuroligin 1
8084146	FXR1	"fragile X mental retardation, autosomal homolog 1 "
8084303	EIF2B5	"eukaryotic translation initiation factor 2B, subunit 5 epsilon, 82kDa "
8084397	ECE2	endothelin converting enzyme 2
8084496	CHRD	chordin
8084794	IL1RAP	interleukin 1 receptor accessory protein
8084880	HES1	"hairy and enhancer of split 1, (Drosophila) "
8085138	OXTR	oxytocin receptor
8085206	CAMK1	calcium/calmodulin-dependent protein kinase I
8085311	ATP2B2	"ATPase, Ca++ transporting, plasma membrane 2 "
8085475	WNT7A	"wingless-type MMTV integration site family, member 7A "
8085914	SLC4A7	solute carrier family 4, sodium bicarbonate cotransporter, member 7

8085946	EOMES	eomesodermin homolog (Xenopus laevis)
8086517	CDCP1	CUB domain containing protein 1
8087405	GPX1	glutathione peroxidase 1
8087409	RHOA	"ras homolog gene family, member A "
8087907	SEMA3G	sema domain, immunoglobulin domain (Ig), short basic domain, secreted, (semaphorin) 3G
8088180	WNT5A	"wingless-type MMTV integration site family, member 5A "
8088384	PDHB	pyruvate dehydrogenase (lipoamide) beta
8088485	FEZF2	FEZ family zinc finger 2
8088919	ROBO1	"roundabout, axon guidance receptor, homolog 1 (Drosophila) "
8089082	DCBLD2	discoidin, CUB and LCCL domain containing 2
8089145	ABI3BP	ABI family, member 3 (NESH) binding protein
8089801	GSK3B	glycogen synthase kinase 3 beta
8090044	SEMA5B	"sema domain, seven thrombospondin repeats (type 1 and type 1-like), transmembrane domain (TM) and short cytoplasmic domain, (semaphorin) 5B "
8090395	TXNRD3	thioredoxin reductase 3
8090469	GATA2	GATA binding protein 2
8091032	FOXL2	forkhead box L2
8091103	TFDP2	transcription factor Dp-2 (E2F dimerization partner 2)
8091190	ATR	ataxia telangiectasia and Rad3 related
8091411	TM4SF1	transmembrane 4 L six family member 1
8091422	WWTR1	WW domain containing transcription regulator 1
8091537	IGSF10	"immunoglobulin superfamily, member 10 "
8091678	VEPH1	ventricular zone expressed PH domain homolog 1 (zebrafish)
8091972	MECOM	MDS1 and EVI1 complex locus
8092691	BCL6	B-cell CLL/lymphoma 6
8092849	ATP13A3	ATPase type 13A3
8093053	TFRC	transferrin receptor (p90, CD71)
8093500	TACC3	transforming, acidic coiled-coil containing protein 3
8093852	MSX1	msh homeobox 1
8094030	AFAP1	actin filament associated protein 1
8094278	NCAPG	non-SMC condensin I complex, subunit G
8094301	SLIT2	slit homolog 2 (Drosophila)
8095021	SPATA18	spermatogenesis associated 18 homolog (rat)
8095074	GSX2	GS homeobox 2
8095080	PDGFRA	platelet-derived growth factor receptor, alpha polypeptide
8095110	KIT	v-kit Hardy-Zuckerman 4 feline sarcoma viral oncogene homolog
8095262	REST	RE1-silencing transcription factor
8095545	RUFY3	RUN and FYVE domain containing 3
8095585	SLC4A4	solute carrier family 4, sodium bicarbonate cotransporter, member 4
8095870	CCNG2	cyclin G2
8096004	BMP2K	BMP2 inducible kinase
8096050	FGF5	fibroblast growth factor 5
8096070	BMP3	bone morphogenetic protein 3
8096098	COPS4	COP9 constitutive photomorphogenic homolog subunit 4 (Arabidopsis)

8096160	ARHGAP24	Rho GTPase activating protein 24
8096461	ATOH1	atonal homolog 1 (Drosophila)
8096511	BMPR1B	"bone morphogenetic protein receptor, type IB "
8096635	NFKB1	nuclear factor of kappa light polypeptide gene enhancer in B-cells 1
8097256	FGF2	fibroblast growth factor 2 (basic)
8097262	SPATA5	spermatogenesis associated 5
8097282	SPRY1	sprouty homolog 1, antagonist of FGF signaling (Drosophila)
8097356	PLK4	polo-like kinase 4 (Drosophila)
8097480	NAA15	"N(alpha)-acetyltransferase 15, NatA auxiliary subunit "
8097553	IL15	interleukin 15
8097657	SMAD1	SMAD family member 1
8097687	POU4F2	POU class 4 homeobox 2
8097782	RPS3A	ribosomal protein S3A
8098006	GLRB	glycine receptor, beta
8098571	HELT	HES/HEY-like transcription factor
8098576	SLC25A4	solute carrier family 25 (mitochondrial carrier; adenine nucleotide translocator), member 4
8098611	TLR3	toll-like receptor 3
8098880	CTBP1	C-terminal binding protein 1
8099132	CYTL1	cytokine-like 1
8099259	AFAP1	actin filament associated protein 1
8099524	LDB2	LIM domain binding 2
8099633	PPARGC1A	"peroxisome proliferator-activated receptor gamma, coactivator 1 alpha "
8099965	ACOT7	acyl-CoA thioesterase 7
8101507	NKX6-1	NK6 homeobox 1
8102560	MAD2L1	MAD2 mitotic arrest deficient-like 1 (yeast)
8102655	BBS7	Bardet-Biedl syndrome 7
8102789	TERF1	telomeric repeat binding factor (NIMA-interacting) 1
8103728	HMGB2	high-mobility group box 2
8103822	VEGFC	vascular endothelial growth factor C
8103922	CASP3	"caspase 3, apoptosis-related cysteine peptidase "
8104079	FAT1	FAT tumor suppressor homolog 1 (Drosophila)
8104234	TRIP13	thyroid hormone receptor interactor 13
8104369	SRD5A1	"steroid-5-alpha-reductase, alpha polypeptide 1 (3-oxo-5 alpha-steroid delta 4-dehydrogenase alpha 1) "
8104449	CCT5	chaperonin containing TCP1, subunit 5 (epsilon)
8104901	IL7R	interleukin 7 receptor
8104912	SKP2	S-phase kinase-associated protein 2 (p45)
8104930	SLC1A3	"solute carrier family 1 (glial high affinity glutamate transporter), member 3 "
8105067	PTGER4	prostaglandin E receptor 4 (subtype EP4)
8105267	ITGA2	"integrin, alpha 2 (CD49B, alpha 2 subunit of VLA-2 receptor) "
8105302	FST	follistatin
8105523	KIF2A	kinesin heavy chain member 2A
8105828	CCNB1	cyclin B1
8105862	CDK7	cyclin-dependent kinase 7

8106098	MAP1B	microtubule-associated protein 1B
8106210	RGNEF	190 kDa guanine nucleotide exchange factor
8106730	XRCC4	X-ray repair complementing defective repair in Chinese hamster cells 4
8106923	NR2F1	nuclear receptor subfamily 2, group F, member 1
8107330	APC	adenomatous polyposis coli
8107470	PTMA	prothymosin, alpha
8107613	SNX2	sorting nexin 2
8107646	PRDM6	PR domain containing 6
8108238	SMAD5	SMAD family member 5
8108370	EGR1	early growth response 1
8108861	NDFIP1	Nedd4 family interacting protein 1
8108949	POU4F3	POU class 4 homeobox 3
8109086	ADRB2	"adrenergic, beta-2-, receptor, surface "
8109120	AFAP1L1	actin filament associated protein 1-like 1
8109194	SLC26A2	"solute carrier family 26 (sulfate transporter), member 2 "
8109639	PTTG1	pituitary tumor-transforming 1 // pituitary tumor-transforming 1
8109697	CCNG1	cyclin G1
8109969	TLX3	T-cell leukemia homeobox 3
8109981	FGF18	fibroblast growth factor 18
8110084	MSX2	msh homeobox 2
8110090	SFXN1	sideroflexin 1
8110569	SQSTM1	sequestosome 1
8110783	TERT	telomerase reverse transcriptase
8110932	SEMA5A	"sema domain, seven thrombospondin repeats (type 1 and type 1-like), transmembrane domain (TM) and short cytoplasmic domain, (semaphorin) 5A "
8111677	LIFR	leukemia inhibitory factor receptor alpha
8111772	DAB2	"disabled homolog 2, mitogen-responsive phosphoprotein (Drosophila) "
8112139	IL6ST	"interleukin 6 signal transducer (gp130, oncostatin M receptor) "
8112202	PLK2	polo-like kinase 2
8112772	AP3B1	"adaptor-related protein complex 3, beta 1 subunit "
8112940	SSBP2	single-stranded DNA binding protein 2
8113010	CCNH	cyclin H
8113039	MEF2C	myocyte enhancer factor 2C
8113250	ERAP1	endoplasmic reticulum aminopeptidase 1
8113433	EFNA5	ephrin-A5
8113573	TSSK1B	testis-specific serine kinase 1B
8113666	SEMA6A	"sema domain, transmembrane domain (TM), and cytoplasmic domain, (semaphorin) 6A "
8114010	IRF1	interferon regulatory factor 1
8114158	PPP2CA	"protein phosphatase 2, catalytic subunit, alpha isozyme "
8114245	NEUROG1	neurogenin 1
8114583	SRA1	steroid receptor RNA activator 1
8114612	CD14	CD14 molecule
8114691	HDAC3	histone deacetylase 3
8114805	FGF1	fibroblast growth factor 1 (acidic)

8114814	NR3C1	nuclear receptor subfamily 3, group C, member 1 (glucocorticoid receptor)
8115147	CD74	"CD74 molecule, major histocompatibility complex, class II invariant chain "
8115158	RPS14	ribosomal protein S14
8115234	ANXA6	annexin A6
8115327	SPARC	"secreted protein, acidic, cysteine-rich (osteonectin) "
8115691	SLIT3	slit homolog 3 (Drosophila)
8115814	SH3PXD2B	SH3 and PX domains 2B
8115840	NKX2-5	"NK2 transcription factor related, locus 5 (Drosophila) "
8116051	DBN1	drebrin 1
8116070	PDLIM7	PDZ and LIM domain 7 (enigma)
8116162	PROP1	PROP paired-like homeobox 1
8116402	MAPK9	mitogen-activated protein kinase 9
8116591	FOXC1	forkhead box C1
8116649	TUBB2A	"tubulin, beta 2A "
8116653	TUBB2A	"tubulin, beta 2A "
8116740	RREB1	ras responsive element binding protein 1
8116780	DSP	desmoplakin
8116921	EDN1	endothelin 1
8116983	CD83	CD83 molecule
8116998	JARID2	"jumonji, AT rich interactive domain 2 "
8117120	ID4	"inhibitor of DNA binding 4, dominant negative helix-loop-helix protein "
8117165	SOX4	SRY (sex determining region Y)-box 4
8117900	DDR1	discoidin domain receptor tyrosine kinase 1
8117995	TUBB	"tubulin, beta "
8118726	GGNBP1	gametogenetin binding protein 1
8118945	PPARD	peroxisome proliferator-activated receptor delta
8119080	SRSF3	serine/arginine-rich splicing factor 3
8119088	CDKN1A	cyclin-dependent kinase inhibitor 1A (p21, Cip1)
8119466	MDFI	MyoD family inhibitor
8119503	TAF8	"TAF8 RNA polymerase II, TATA box binding protein (TBP)-associated factor, 43kDa "
8119712	SRF	serum response factor (c-fos serum response element-binding transcription factor)
8119898	VEGFA	vascular endothelial growth factor A
8119974	SLC29A1	solute carrier family 29 (nucleoside transporters), member 1
8120043	RUNX2	runt-related transcription factor 2
8120215	PAQR8	progestin and adipoQ receptor family member VIII
8120585	SMAP1	small ArfGAP 1
8120783	MYO6	myosin VI
8120967	NT5E	5'-nucleotidase, ecto (CD73)
8121209	POU3F2	POU class 3 homeobox 2
8121257	PRDM1	PR domain containing 1, with ZNF domain
8121365	FOXO3	forkhead box O3
8121704	NUS1	nuclear undecaprenyl pyrophosphate synthase 1 homolog (S. cerevisiae)
8121850	HEY2	hairy/enhancer-of-split related with YRPW motif 2

8122099	ENPP1	ectonucleotide pyrophosphatase/phosphodiesterase 1
8122176	TCF21	transcription factor 21
8122242	PEX7	peroxisomal biogenesis factor 7
8122334	CCRL1	chemokine (C-C motif) receptor-like 1
8123315	QKI	"quaking homolog, KH domain RNA binding (mouse) "
8123494	FAM120B	family with sequence similarity 120B
8123644	TUBB2A	"tubulin, beta 2A "
8123651	TUBB2B	"tubulin, beta 2B "
8124280	FAM65B	"family with sequence similarity 65, member B "
8124654	GABBR1	"gamma-aminobutyric acid (GABA) B receptor, 1 "
8124848	IER3	immediate early response 3
8124862	CDSN	corneodesmosin
8124868	CCHCR1	coiled-coil alpha-helical rod protein 1
8124889	POU5F1	POU class 5 homeobox 1
8125295	ATF6B	activating transcription factor 6 beta
8125383	NOTCH4	Notch homolog 4 (Drosophila)
8125545	HLA-DOA	"major histocompatibility complex, class II, DO alpha "
8125568	COL11A2	"collagen, type XI, alpha 2 "
8125713	TAPBP	TAP binding protein (tapasin)
8125941	SRPK1	SFRS protein kinase 1
8126102	MDGA1	MAM domain containing glycosylphosphatidylinositol anchor 1
8126474	MEA1	male-enhanced antigen 1
8126574	YIPF3	"Yip1 domain family, member 3 "
8127031	MCM3	minichromosome maintenance complex component 3
8127446	COL9A1	"collagen, type IX, alpha 1 "
8127841	PGM3	phosphoglucomutase 3
8128429	CCNC	cyclin C
8128669	OSTM1	osteopetrosis associated transmembrane protein 1
8128991	LAMA4	"laminin, alpha 4 "
8129562	CTGF	connective tissue growth factor
8129937	CITED2	"Cbp/p300-interacting transactivator, with Glu/Asp-rich carboxy-terminal domain, 2 "
8130211	SYNE1	"spectrin repeat containing, nuclear envelope 1 "
8130505	EZR	ezrin
8130556	SOD2	"superoxide dismutase 2, mitochondrial "
8130939	DLL1	delta-like 1 (Drosophila)
8131087	UNCX	UNC homeobox
8131253	FOXK1	forkhead box K1
8131374	AIMP2	aminoacyl tRNA synthetase complex-interacting multifunctional protein 2
8131475	C1GALT1	"core 1 synthase, glycoprotein-N-acetylgalactosamine 3-beta-galactosyltransferase, 1 "
8131550	SCIN	scinderin
8131583	BZW2	basic leucine zipper and W2 domains 2
8131631	HDAC9	histone deacetylase 9
8131803	IL6	"interleukin 6 (interferon, beta 2) "

8131844	GPNMB	glycoprotein (transmembrane) nmb
8131996	CREB5	cAMP responsive element binding protein 5
8132036	WIPF3	"WAS/WASL interacting protein family, member 3 "
8132218	BBS9	Bardet-Biedl syndrome 9
8132805	VWC2	von Willebrand factor C domain containing 2
8132860	EGFR	"epidermal growth factor receptor (erythroblastic leukemia viral (v-erb-b) oncogene homolog, avian) "
8134117	FZD1	frizzled homolog 1 (Drosophila)
8134263	COL1A2	collagen, type I, alpha 2
8134339	PEG10	paternally expressed 10
8134407	DLX6	distal-less homeobox 6
8134907	EPO	erythropoietin
8135069	SERPINE1	serpin peptidase inhibitor, clade E (nexin, plasminogen activator inhibitor type 1), member 1
8135149	SH2B2	SH2B adaptor protein 2
8135218	LRRC17	leucine rich repeat containing 17
8135277	MLL5	"myeloid/lymphoid or mixed-lineage leukemia 5 (trithorax homolog, Drosophila) "
8135514	IFRD1	interferon-related developmental regulator 1
8135594	CAV1	"caveolin 1, caveolae protein, 22kDa "
8135763	WNT16	wingless-type MMTV integration site family, member 16
8136080	SMO	smoothened homolog (Drosophila)
8137865	GNA12	guanine nucleotide binding protein (G protein) alpha 12
8137979	ACTB	actin, beta
8138189	RPA3	replication protein A3, 14kDa
8138442	TWIST1	twist homolog 1 (Drosophila)
8138602	DFNA5	"deafness, autosomal dominant 5 "
8139087	SFRP4	secreted frizzled-related protein 4
8139207	INHBA	"inhibin, beta A "
8139212	GLI3	GLI family zinc finger 3
8139430	PURB	purine-rich element binding protein B
8139488	IGFBP3	insulin-like growth factor binding protein 3
8140319	HIP1	huntingtin interacting protein 1
8140398	YWHAG	"tyrosine 3-monooxygenase/tryptophan 5-monooxygenase activation protein, gamma polypeptide "
8140556	HGF	hepatocyte growth factor (hepapoietin A; scatter factor)
8140650	SEMA3E	"sema domain, immunoglobulin domain (Ig), short basic domain, secreted, (semaphorin) 3E "
8140668	SEMA3A	"sema domain, immunoglobulin domain (Ig), short basic domain, secreted, (semaphorin) 3A "
8140686	SEMA3D	"sema domain, immunoglobulin domain (Ig), short basic domain, secreted, (semaphorin) 3D "
8140955	CDK6	cyclin-dependent kinase 6
8141016	TFPI2	tissue factor pathway inhibitor 2
8141050	RPS3A	ribosomal protein S3A
8141140	DLX5	distal-less homeobox 5
8141222	RPS3A	ribosomal protein S3A
8141241	SMURF1	SMAD specific E3 ubiquitin protein ligase 1
8141380	ZNF3	zinc finger protein 3
8141466	GPC2	glypican 2

8141950	RELN	reelin
8142036	SRPK2	SFRS protein kinase 2
8142468	TPM3	tropomyosin 3
8142471	WNT2	wingless-type MMTV integration site family member 2
8142580	FEZF1	FEZ family zinc finger 1
8142747	PAX4	paired box 4
8142981	PODXL	podocalyxin-like
8143188	CREB3L2	cAMP responsive element binding protein 3-like 2
8143772	RARRES2	retinoic acid receptor responder (tazarotene induced) 2
8143850	CDK5	cyclin-dependent kinase 5
8143919	SMARCD3	"SWI/SNF related, matrix associated, actin dependent regulator of chromatin, subfamily d, member 3 "
8144078	SHH	sonic hedgehog homolog (Drosophila)
8144112	MNX1	motor neuron and pancreas homeobox 1
8144267	CLN8	"ceroid-lipofuscinosis, neuronal 8 (epilepsy, progressive with mental retardation) "
8144279	CLN8	"ceroid-lipofuscinosis, neuronal 8 (epilepsy, progressive with mental retardation) "
8144643	GATA4	GATA binding protein 4
8144758	ZDHHC2	zinc finger, DHHC-type containing 2
8145055	BMP1	bone morphogenetic protein 1
8145470	DPYSL2	dihydropyrimidinase-like 2
8145490	PTK2B	PTK2B protein tyrosine kinase 2 beta
8145736	NRG1	neuregulin 1
8145865	GPR124	G protein-coupled receptor 124
8145954	TACC1	transforming, acidic coiled-coil containing protein 1
8146000	ADAM9	ADAM metallopeptidase domain 9 (meltrin gamma)
8146216	VDAC3	voltage-dependent anion channel 3
8146357	MCM4	minichromosome maintenance complex component 4
8146462	SOX17	SRY (sex determining region Y)-box 17
8146500	LYN	v-yes-1 Yamaguchi sarcoma viral related oncogene homolog
8146645	BHLHE22	"basic helix-loop-helix family, member e22 "
8146914	TERF1	telomeric repeat binding factor (NIMA-interacting) 1
8147049	FABP5	fatty acid binding protein 5 (psoriasis-associated)
8147461	SDC2	syndecan 2
8147516	MATN2	matrilin 2
8148317	MYC	v-myc myelocytomatosis viral oncogene homolog (avian)
8148559	C8orf55	chromosome 8 open reading frame 55
8148572	LY6E	lymphocyte antigen 6 complex, locus E
8148597	ZFP41	zinc finger protein 41 homolog (mouse)
8149330	CTSB	cathepsin B
8149749	TNFRSF10D	tumor necrosis factor receptor superfamily, member 10d, decoy with truncated death domain
8149820	NKX2-6	"NK2 transcription factor related, locus 6 (Drosophila) "
8149825	STC1	stanniocalcin 1
8149927	CLU	clusterin
8149955	PBK	PDZ binding kinase

8150287	WHSC1L1	Wolf-Hirschhorn syndrome candidate 1-like 1
8150318	FGFR1	fibroblast growth factor receptor 1
8150428	SFRP1	secreted frizzled-related protein 1
8150491	MYST3	MYST histone acetyltransferase (monocytic leukemia) 3
8150592	CEBPD	CCAAT/enhancer binding protein (C/EBP), delta
8150599	PRKDC	"protein kinase, DNA-activated, catalytic polypeptide "
8150698	SNAI2	snail homolog 2 (Drosophila)
8150818	TCEA1	"transcription elongation factor A (SII), 1 "
8150830	LYPLA1	lysophospholipase I
8151310	EYA1	eyes absent homolog 1 (Drosophila)
8151711	NBN	nibrin
8151768	RUNX1T1	"runt-related transcription factor 1; translocated to, 1 (cyclin D-related) "
8151906	GDF6	growth differentiation factor 6
8152215	KLF10	Kruppel-like factor 10
8152280	LRP12	low density lipoprotein receptor-related protein 12
8152297	ANGPT1	angiopoietin 1
8152491	EXT1	exostosin 1
8152512	TNFRSF11B	tumor necrosis factor receptor superfamily, member 11b
8153175	TRAPPC9	trafficking protein particle complex 9
8153449	EEF1D	eukaryotic translation elongation factor 1 delta (guanine nucleotide exchange protein)
8153457	EEF1D	eukaryotic translation elongation factor 1 delta (guanine nucleotide exchange protein)
8153497	SCRIB	scribbled homolog (Drosophila)
8153828	FOXH1	forkhead box H1
8154043	DMRT3	doublesex and mab-3 related transcription factor 3
8154100	VLDLR	very low density lipoprotein receptor
8154178	JAK2	Janus kinase 2
8154316	UHRF2	ubiquitin-like with PHD and ring finger domains 2
8154656	DMRTA1	DMRT-like family A1
8155096	CREB3	cAMP responsive element binding protein 3
8155849	ANXA1	annexin A1
8155883	OSTF1	osteoclast stimulating factor 1
8156134	NTRK2	"neurotrophic tyrosine kinase, receptor, type 2 "
8156290	CKS2	CDC28 protein kinase regulatory subunit 2
8156309	GADD45G	"growth arrest and DNA-damage-inducible, gamma "
8156783	COL15A1	"collagen, type XV, alpha 1 "
8156826	TGFBR1	"transforming growth factor, beta receptor 1 "
8157487	PAPPA	"pregnancy-associated plasma protein A, pappalysin 1 "
8157516	TRIM32	tripartite motif-containing 32
8157524	TLR4	toll-like receptor 4
8157922	LMX1B	"LIM homeobox transcription factor 1, beta "
8158112	CDK9	cyclin-dependent kinase 9
8158269	ODF2	outer dense fiber of sperm tails 2
8158424	LRRC8A	"leucine rich repeat containing 8 family, member A "

8158725 ABL1	c-abl oncogene 1, non-receptor tyrosine kinase
8158890 NTNG2	netrin G2
8159127 RXRA	"retinoid X receptor, alpha "
8159318 GPSM1	"G-protein signaling modulator 1 (AGS3-like, C. elegans) "
8159850 VLDLR	very low density lipoprotein receptor
8160238 PSIP1	PC4 and SFRS1 interacting protein 1
8160441 CDKN2A	"cyclin-dependent kinase inhibitor 2A (melanoma, p16, inhibits CDK4) "
8160452 CDKN2B	"cyclin-dependent kinase inhibitor 2B (p15, inhibits CDK4) "
8160647 BAG1	BCL2-associated athanogene
8160663 AQP7	aquaporin 7
8160670 AQP3	aquaporin 3 (Gill blood group)
8160914 VCP	valosin-containing protein
8161211 PAX5	paired box 5
8161255 SHB	Src homology 2 domain containing adaptor protein B
8161906 GNAQ	"guanine nucleotide binding protein (G protein), q polypeptide "
8162236 SEMA4D	"sema domain, immunoglobulin domain (Ig), transmembrane domain (TM) and short cytoplasmic domain, (semaphorin) 4D "
8162472 BARX1	BARX homeobox 1
8162533 PTCH1	patched 1
8162759 TBC1D2	TBC1 domain family, member 2
8162940 ABCA1	"ATP-binding cassette, sub-family A (ABC1), member 1 "
8163002 KLF4	Kruppel-like factor 4 (gut)
8163202 SVEP1	sushi, von Willebrand factor type A, EGF and pentraxin domain containing 1
8163444 ZFP37	zinc finger protein 37 homolog (mouse)
8163672 PAPPA	"pregnancy-associated plasma protein A, pappalysin 1 "
8163733 CDK5RAP2	CDK5 regulatory subunit associated protein 2
8163936 LHX6	LIM homeobox 6
8164013 STRBP	spermatid perinuclear RNA binding protein
8164077 NR5A1	"nuclear receptor subfamily 5, group A, member 1 "
8164269 ENG	endoglin
8164781 TSC1	tuberous sclerosis 1
8165038 SOHLH1	spermatogenesis and oogenesis specific basic helix-loop-helix 1
8165217 NOTCH1	"Notch homolog 1, translocation-associated (Drosophila) "
8165309 EDF1	endothelial differentiation-related factor 1
8166079 EGFL6	"EGF-like-domain, multiple 6 "
8166266 NHS	Nance-Horan syndrome (congenital cataracts and dental anomalies)
8166455 PRDX4	peroxiredoxin 4
8167347 SUV39H1	suppressor of variegation 3-9 homolog 1 (Drosophila)
8167407 ERAS	ES cell expressed Ras
8167973 HEPH	hephaestin
8167998 AR	androgen receptor
8168045 EFNB1	ephrin-B1
8168205 FOXO4	forkhead box O4
8168472 ATP7A	"ATPase, Cu++ transporting, alpha polypeptide "

8168691	DIAPH2	diaphanous homolog 2 (Drosophila)
8168794	CENPI	centromere protein I
8169061	PLP1	proteolipid protein 1
8169640	SLC25A5	solute carrier family 25 (mitochondrial carrier; adenine nucleotide translocator), member 5
8170119	FHL1	four and a half LIM domains 1
8170753	SRPK3	SFRS protein kinase 3
8170921	PLXNA3	plexin A3
8171867	ARX	aristaless related homeobox
8172520	TFE3	transcription factor binding to IGHM enhancer 3
8172905	HSD17B10	hydroxysteroid (17-beta) dehydrogenase 10
8172914	HUWE1	"HECT, UBA and WWE domain containing 1 "
8173299	EDA2R	ectodysplasin A2 receptor
8173673	ATRX	alpha thalassemia/mental retardation syndrome X-linked
8174201	BEX1	"brain expressed, X-linked 1 "
8174253	MORF4L2	mortality factor 4 like 2
8174361	TSC22D3	TSC22 domain family, member 3
8174675	SLC25A5	solute carrier family 25 (mitochondrial carrier; adenine nucleotide translocator), member 5
8174985	SMARCA1	"SWI/SNF related, matrix associated, actin dependent regulator of chromatin, subfamily a, member 1 "
8175177	MBNL3	muscleblind-like 3 (Drosophila)
8175528	SOX3	SRY (sex determining region Y)-box 3
8176276	ATRX	alpha thalassemia/mental retardation syndrome X-linked
8177462	CDK7	cyclin-dependent kinase 7
8177858	TUBB	"tubulin, beta "
8177867	DDR1	discoidin domain receptor tyrosine kinase 1
8178298	GABBR1	"gamma-aminobutyric acid (GABA) B receptor, 1 "
8178435	IER3	immediate early response 3
8178442	CDSN	corneodesmosin
8178448	CCHCR1	coiled-coil alpha-helical rod protein 1
8178470	POU5F1	POU class 5 homeobox 1
8178727	ATF6B	activating transcription factor 6 beta
8178897	COL11A2	"collagen, type XI, alpha 2 "
8178977	TAPBP	TAP binding protein (tapasin)
8179174	TUBB	"tubulin, beta "
8179184	DDR1	discoidin domain receptor tyrosine kinase 1
8179595	GABBR1	"gamma-aminobutyric acid (GABA) B receptor, 1 "
8179704	IER3	immediate early response 3
8179716	CDSN	corneodesmosin
8179719	POU5F1	POU class 5 homeobox 1
8180093	HLA-DOA	"major histocompatibility complex, class II, DO alpha "
8180105	COL11A2	"collagen, type XI, alpha 2 "
8180166	TAPBP	TAP binding protein (tapasin)
