# Supplementary material for: Characterization of proliferation, differentiation potential, and gene expression among clonal cultures of human dental pulp cells
Source: Hum Cell. 2020 Mar 16;33(3):490–501. doi: 10.1007/s13577-020-00327-9 (PMC7324427; doi:10.1007/s13577-020-00327-9)
Supplement: Supplementary file 3 — Supplementary file3 (PDF 10 kb) [file 13577_2020_327_MOESM3_ESM.pdf]

characterization of proliferation, differentiation potential, and gene expression among clonal cultures of human dental pulp cells

Tomoko Kobayashi[1,2], Daisuke Torii [3], Takanori Iwata [2], Yuichi Izumi [2,4], Masanori Nasu [1], Takeo W. Tsutsui[3]

1 Research Center for Odontology, School of Life Dentistry at Tokyo, The Nippon Dental University
2 Department of Periodontology, Graduate School of Medical and Dental Sciences, Tokyo Medical and Dental University (TMDU)
3 Department of Pharmacology, School of Life Dentistry at Tokyo, The Nippon Dental University
4 Oral Care Perio Center, Southern TOHOKU General Hospital, Southern TOHOKU Research Institute for Neuroscience

Corresponding author: Takeo W. Tsutsui (ryuryu@tky.ndu.ac.jp)

Supplemental Table S3. Sources of the probeset list of gene groups related to 'stemness or differentiation'.
Genes were extracted from the database of listed Ingenuity Pathway Analysis (IPA) biofunctions, Gene Ontology, and published scientific literature.

IPA BioFunction
activation of mesenchymal stem cells
activation of mesenchymal stem cells list
adipogenesis of mesenchymal stem cells
adipogenesis of mesenchymal stem cells list
chondrogenesis of mesenchymal stem cells
chondrogenesis of mesenchymal stem cells list
Differentiation of adipose mesenchymal stem cells
Differentiation of adipose mesenchymal stem cells list
Differentiation of mesenchymal stem cells
Differentiation of mesenchymal stem cells list
lifespan of mesenchymal stem cells
lifespan of mesenchymal stem cells list
mineralization of mesenchymal stem cells
mineralization of mesenchymal stem cells list
pluripotency of mesenchymal stem cells
pluripotency of mesenchymal stem cells list
proliferation of mesenchymal stem cells
proliferation of mesenchymal stem cells list
self-renewal of mesenchymal stem cells
self-renewal of mesenchymal stem cells list

Gene Ontology
genes which include "stem cell" in its GO_Biological process_Term
genes which include "ossification" in its GO_Biological process_Term
genes which include "differentiation" in its GO_Biological process_Term

literature (DOI)
DOI: 10.1016/s0006-291x(02)00661-7
DOI: 10.1016/s0014-4827(02)00012-5
DOI: 10.1016/s0014-4827(03)00349-5
DOI: 10.1177/154405910408300802
DOI: 10.1111/j.1601-6343.2005.00331.x
DOI: 10.1111/j.1600-0722.2005.00221.x
DOI: 10.1634/stemcells.2005-0604
DOI: 10.1634/stemcells.2006-0161
DOI: 10.1152/physiolgenomics.00197.2006
DOI: 10.1016/j.joen.2007.02.009
DOI: 10.1634/stemcells.2007-0225
DOI: 10.1016/j.bbrc.2007.10.149
DOI: 10.1177/154405910808700206
DOI: 10.1177/154405910808700312
DOI: 10.1186/1471-2164-9-340
DOI: 10.1016/j.joen.2008.06.014
DOI: 10.1016/j.archoralbio.2008.10.003
DOI: 10.1111/j.1365-263X.2008.00964.x
DOI: 10.1177/0022034509342363
DOI: 10.1089/ten.TEA.2009.0307
DOI: 10.1016/j.joen.2009.07.005
DOI: 10.1016/j.cytogfr.2009.10.012
DOI: 10.1089/scd.2009.0492
DOI: 10.1177/0022034510364487
DOI: 10.1111/j.1747-4477.2009.00188.x
DOI: 10.1038/nature09347
DOI: 10.1089/scd.2010.0353
DOI: 10.1038/nature09531
DOI: 10.1016/j.bbamcr.2011.01.022
DOI: 10.1016/j.archoralbio.2011.02.006
DOI: 10.1073/pnas.1100816108
DOI: 10.1111/j.1749-6632.2011.06234.x
DOI: 10.1016/j.molcel.2012.05.022
