# Supplementary material for: Characterization of proliferation, differentiation potential, and gene expression among clonal cultures of human dental pulp cells
Source: Hum Cell. 2020 Mar 16;33(3):490–501. doi: 10.1007/s13577-020-00327-9 (PMC7324427; doi:10.1007/s13577-020-00327-9)
Supplement: Supplementary file 4 — Supplementary file4 (PDF 18 kb) [file 13577_2020_327_MOESM4_ESM.pdf]

characterization of proliferation, differentiation potential, and gene expression among clonal cultures of human dental pulp cells

Tomoko Kobayashi[1,2], Daisuke Torii [3], Takanori Iwata [2], Yuichi Izumi [2,4], Masanori Nasu [1], Takeo W. Tsutsui[3]

1 Research Center for Odontology, School of Life Dentistry at Tokyo, The Nippon Dental University
2 Department of Periodontology, Graduate School of Medical and Dental Sciences, Tokyo Medical and Dental University (TMDU)
3 Department of Pharmacology, School of Life Dentistry at Tokyo, The Nippon Dental University
4 Oral Care Perio Center, Southern TOHOKU General Hospital, Southern TOHOKU Research Institute for Neuroscience

Corresponding author: Takeo W. Tsutsui (ryuryu@tky.ndu.ac.jp)

Supplemental Table S4. DNA microarray data of 95 selected probesets related to multipotency and 'stemness or differentiation'.
Signal intensity values <100 are shaded in grey; values >1000 are represented in red letters. Ratios <0.67 are shaded in blue; ratios <0.83 are shaded in light blue.
Ratios >1.5 are shaded in pink; ratios >1.2 are shaded in light pink.

ProbeSet ID	Gene_ Symbol	cl21_ signal	cl4_ signal	cl35_ signal	cl29_ signal	cl38_ signal	ratio 21/29	ratio 4/29	ratio 35/29	ratio 29/38	ratio 4/38	ratio 21/38	ratio 35/38	Location	Type(s)
8140650	*SEMA3E*	24	48	68	244	263	0.1	0.2	0.3	0.9	0.2	0.1	0.3	Extracellular Space	other
7973974	*PAX9*	587	610	130	1559	2090	0.4	0.4	0.1	0.7	0.3	0.3	0.1	Nucleus	transcription regulator
8140686	*SEMA3D*	496	699	920	1634	2613	0.3	0.4	0.6	0.6	0.3	0.2	0.4	Extracellular Space	other
8091537	*IGSF10*	34	50	43	74	189	0.5	0.7	0.6	0.4	0.3	0.2	0.2	unknown	other
8046922	*COL3A1*	2071	1648	2123	3354	4465	0.6	0.5	0.6	0.8	0.4	0.5	0.5	Extracellular Space	other
7961798	*SOX5*	80	71	110	139	171	0.6	0.5	0.8	0.8	0.4	0.5	0.6	Nucleus	transcription regulator
7932254	*ITGA8*	4644	4130	2372	5718	6662	0.8	0.7	0.4	0.9	0.6	0.7	0.4	Plasma Membrane	other
8095545	*RUFY3*	275	426	426	445	787	0.6	1.0	1.0	0.6	0.5	0.3	0.5	Plasma Membrane	other
8139212	*GLI3*	345	827	667	905	1056	0.4	0.9	0.7	0.9	0.8	0.3	0.6	Nucleus	transcription regulator
8138442	*TWIST1*	545	885	738	1074	1185	0.5	0.8	0.7	0.9	0.7	0.5	0.6	Nucleus	transcription regulator
7973530	*PCK2*	1058	774	608	1220	1253	0.9	0.6	0.5	1.0	0.6	0.8	0.5	Cytoplasm	kinase
7943620	*ATM*	505	568	623	640	845	0.8	0.9	1.0	0.8	0.7	0.6	0.7	Nucleus	kinase
8076331	*TOB2*	559	865	886	919	1062	0.6	0.9	1.0	0.9	0.8	0.5	0.8	Nucleus	other
8145865	*GPR124*	1269	1541	1335	1691	1842	0.8	0.9	0.8	0.9	0.8	0.7	0.7	Plasma Membrane	G-protein coupled receptor
7935968	*LDB1*	2160	2369	2599	2815	3151	0.8	0.8	0.9	0.9	0.8	0.7	0.8	Nucleus	transcription regulator
8042439	*ANTXR1*	1588	1379	1784	1942	1950	0.8	0.7	0.9	1.0	0.7	0.8	0.9	Plasma Membrane	transmembrane receptor
8134263	*COL1A2*	8190	8134	8960	8973	10817	0.9	0.9	1.0	0.8	0.8	0.8	0.8	Extracellular Space	other
8001306	*SIAH1*	297	296	297	312	355	1.0	1.0	1.0	0.9	0.8	0.8	0.8	Nucleus	enzyme
8109981	*FGF18*	151	151	148	156	176	1.0	1.0	0.9	0.9	0.9	0.9	0.8	Extracellular Space	growth factor
8160663	*AQP7*	119	119	119	123	124	1.0	1.0	1.0	1.0	1.0	1.0	1.0	Plasma Membrane	transporter
8137979	*ACTB*	10177	9947	9969	9842	9529	1.0	1.0	1.0	1.0	1.0	1.1	1.0	Cytoplasm	other
7923027	*GLRX2*	215	207	207	207	196	1.0	1.0	1.0	1.1	1.1	1.1	1.1	Cytoplasm	enzyme
8050215	*YWHAQ*	6519	6527	6327	6300	6039	1.0	1.0	1.0	1.0	1.1	1.1	1.0	Cytoplasm	other
8109194	*SLC26A2*	336	361	349	323	322	1.0	1.1	1.1	1.0	1.1	1.0	1.1	Plasma Membrane	transporter
8061136	*PTMA*	11139	11215	11203	10603	10185	1.1	1.1	1.1	1.0	1.1	1.1	1.1	Nucleus	other
7991332	*MESP1*	280	280	293	279	254	1.0	1.0	1.0	1.1	1.1	1.1	1.2	Nucleus	transcription regulator
7947540	*TRAF6*	532	448	449	442	432	1.2	1.0	1.0	1.0	1.0	1.2	1.0	Cytoplasm	enzyme
7949067	*BAD*	412	407	410	406	357	1.0	1.0	1.0	1.1	1.1	1.2	1.1	Cytoplasm	other
7898988	*CLIC4*	6959	7233	6547	6404	6162	1.1	1.1	1.0	1.0	1.2	1.1	1.1	Plasma Membrane	ion channel
7961022	*PTMA*	4342	4899	4342	4131	4051	1.1	1.2	1.1	1.0	1.2	1.1	1.1	Nucleus	other
7954006	*PTMA*	4184	4693	4182	3977	3871	1.1	1.2	1.1	1.0	1.2	1.1	1.1	Nucleus	other
7985983	*NGRN*	618	593	586	545	532	1.1	1.1	1.1	1.0	1.1	1.2	1.1	Nucleus	other
7936661	*PRDX3*	1719	1878	1673	1588	1546	1.1	1.2	1.1	1.0	1.2	1.1	1.1	Cytoplasm	enzyme
8166455	*PRDX4*	935	879	853	841	758	1.1	1.0	1.0	1.1	1.2	1.2	1.1	Cytoplasm	enzyme
8142468	*TPM3*	5250	5175	5091	5080	4338	1.0	1.0	1.0	1.2	1.2	1.2	1.2	Cytoplasm	other
8060850	*BMP2*	131	154	131	131	116	1.0	1.2	1.0	1.1	1.3	1.1	1.1	Extracellular Space	growth factor
8114158	*PPP2CA*	2237	2253	2115	1985	1877	1.1	1.1	1.1	1.1	1.2	1.2	1.1	Cytoplasm	phosphatase
8040142	*CPSF3*	629	577	515	514	489	1.2	1.1	1.0	1.1	1.2	1.3	1.1	Nucleus	enzyme
7940530	*C11orf9*	105	100	101	91	86	1.2	1.1	1.1	1.1	1.2	1.2	1.2	Nucleus	transcription regulator
8138189	*RPA3*	164	158	145	144	128	1.1	1.1	1.0	1.1	1.2	1.3	1.1	Nucleus	other
8027402	*CCNE1*	155	138	134	122	120	1.3	1.1	1.1	1.0	1.2	1.3	1.1	Nucleus	transcription regulator
8018364	*GRB2*	673	704	664	613	556	1.1	1.1	1.1	1.1	1.3	1.2	1.2	Cytoplasm	other
8073733	*NUP50*	166	155	155	144	126	1.2	1.1	1.1	1.1	1.2	1.3	1.2	Nucleus	transporter
8046461	*ZAK*	1357	1190	1184	1063	1002	1.3	1.1	1.1	1.1	1.2	1.4	1.2	Cytoplasm	kinase
7929988	*HPS6*	266	276	243	233	204	1.1	1.2	1.0	1.1	1.4	1.3	1.2	Cytoplasm	other
7913869	*STMN1*	2413	2257	2159	1854	1822	1.3	1.2	1.2	1.0	1.2	1.3	1.2	Cytoplasm	other
8146216	*VDAC3*	3289	2880	2877	2719	2297	1.2	1.1	1.1	1.2	1.3	1.4	1.3	Cytoplasm	ion channel
8103922	*CASP3*	460	416	437	380	322	1.2	1.1	1.2	1.2	1.3	1.4	1.4	Cytoplasm	peptidase
8125941	*SRPK1*	1063	1221	1174	1020	840	1.0	1.2	1.2	1.2	1.5	1.3	1.4	Nucleus	kinase
8109120	*AFAP1L1*	205	147	147	130	128	1.6	1.1	1.1	1.0	1.1	1.6	1.1	unknown	other
8024557	*GNA11*	2647	2034	1883	1703	1660	1.6	1.2	1.1	1.0	1.2	1.6	1.1	Plasma Membrane	enzyme
7920984	*CCT3*	1554	1420	1577	1389	1060	1.1	1.0	1.1	1.3	1.3	1.5	1.5	Cytoplasm	other
7912481	*MAD2L2*	1110	854	853	828	656	1.3	1.0	1.0	1.3	1.3	1.7	1.3	Nucleus	enzyme
8106730	*XRCC4*	162	131	129	123	98	1.3	1.1	1.1	1.2	1.3	1.6	1.3	Nucleus	other
8040070	*SOX11*	388	440	438	354	294	1.1	1.2	1.2	1.2	1.5	1.3	1.5	Nucleus	transcription regulator
7927955	*KIAA1279*	1348	973	932	839	787	1.6	1.2	1.1	1.1	1.2	1.7	1.2	Cytoplasm	enzyme
8024566	---	2226	1871	1569	1468	1354	1.5	1.3	1.1	1.1	1.4	1.6	1.2	#N/A	#N/A
8140398	*YWHAG*	2260	1840	2104	1729	1425	1.3	1.1	1.2	1.2	1.3	1.6	1.5	Cytoplasm	other
8110090	*SFXN1*	1687	1224	1248	1168	931	1.4	1.0	1.1	1.3	1.3	1.8	1.3	Cytoplasm	transporter
8064844	*PCNA*	511	734	525	485	397	1.1	1.5	1.1	1.2	1.8	1.3	1.3	Nucleus	enzyme
8172905	*HSD17B10*	884	768	682	661	507	1.3	1.2	1.0	1.3	1.5	1.7	1.3	Cytoplasm	enzyme
8079153	*ABHD5*	758	913	733	660	526	1.1	1.4	1.1	1.3	1.7	1.4	1.4	Cytoplasm	enzyme
8028719	*DLL3*	116	107	114	95	71	1.2	1.1	1.2	1.3	1.5	1.6	1.6	Extracellular Space	other
7936734	*FGFR2*	93	67	107	63	61	1.5	1.1	1.7	1.0	1.1	1.5	1.7	Plasma Membrane	kinase
8018793	*JMJD6*	641	468	516	397	360	1.6	1.2	1.3	1.1	1.3	1.8	1.4	Plasma Membrane	other
8169640	*SLC25A5*	4068	3118	3323	3076	2154	1.3	1.0	1.1	1.4	1.4	1.9	1.5	Cytoplasm	transporter
7947199	*LGR4*	589	437	468	366	310	1.6	1.2	1.3	1.2	1.4	1.9	1.5	Plasma Membrane	G-protein coupled receptor
8008517	*NME1*	2166	1713	1779	1359	1174	1.6	1.3	1.3	1.2	1.5	1.8	1.5	Nucleus	kinase
8041582	*PKDCC*	210	284	185	157	137	1.3	1.8	1.2	1.1	2.1	1.5	1.4	Cytoplasm	kinase
8093053	*TFRC*	2351	1878	2067	1447	1267	1.6	1.3	1.4	1.1	1.5	1.9	1.6	Plasma Membrane	transporter
8148317	*MYC*	1058	1160	890	824	584	1.3	1.4	1.1	1.4	2.0	1.8	1.5	Nucleus	transcription regulator
7975779	*FOS*	236	303	173	152	146	1.6	2.0	1.1	1.0	2.1	1.6	1.2	Nucleus	transcription regulator
8020455	*GATA6*	393	399	234	233	201	1.7	1.7	1.0	1.2	2.0	2.0	1.2	Nucleus	transcription regulator
8124848	*IER3*	1137	1080	743	670	579	1.7	1.6	1.1	1.2	1.9	2.0	1.3	Cytoplasm	other
8179704	*IER3*	1137	1080	743	670	579	1.7	1.6	1.1	1.2	1.9	2.0	1.3	Cytoplasm	other
7963880	*ITGA7*	232	164	179	127	111	1.8	1.3	1.4	1.1	1.5	2.1	1.6	Plasma Membrane	other
8023497	*ATP8B1*	1714	2367	1636	1317	1068	1.3	1.8	1.2	1.2	2.2	1.6	1.5	Plasma Membrane	transporter
8178435	*IER3*	1578	1484	966	898	746	1.8	1.7	1.1	1.2	2.0	2.1	1.3	Cytoplasm	other
7953100	*FKBP4*	1365	961	1052	786	591	1.7	1.2	1.3	1.3	1.6	2.3	1.8	Nucleus	enzyme
7918323	*SORT1*	4981	3063	2991	2500	1858	2.0	1.2	1.2	1.3	1.6	2.7	1.6	Plasma Membrane	transmembrane receptor
7927964	*SRGN*	6539	5977	4826	4203	2800	1.6	1.4	1.1	1.5	2.1	2.3	1.7	Extracellular Space	other
8099965	*ACOT7*	1349	824	678	648	437	2.1	1.3	1.0	1.5	1.9	3.1	1.6	unknown	other
8139207	*INHBA*	7797	2872	2960	2407	2202	3.2	1.2	1.2	1.1	1.3	3.5	1.3	Extracellular Space	growth factor
8116780	*DSP*	1001	549	443	371	281	2.7	1.5	1.2	1.3	2.0	3.6	1.6	Plasma Membrane	other
8135069	*SERPINE1*	6465	5550	5861	2814	2604	2.3	2.0	2.1	1.1	2.1	2.5	2.3	Extracellular Space	other
8106210	*RGNEF*	254	340	358	243	98	1.0	1.4	1.5	2.5	3.5	2.6	3.7	Cytoplasm	other
8174201	*BEX1*	64	262	75	55	48	1.2	4.8	1.4	1.1	5.4	1.3	1.6	unknown	other
8085138	*OXTR*	3816	1992	2077	1766	680	2.2	1.1	1.2	2.6	2.9	5.6	3.1	Plasma Membrane	G-protein coupled receptor
7973067	*PNP*	626	487	388	198	147	3.2	2.5	2.0	1.3	3.3	4.3	2.6	Nucleus	enzyme
7950307	*UCP2*	1260	410	530	220	215	5.7	1.9	2.4	1.0	1.9	5.9	2.5	Cytoplasm	transporter
8025601	*ICAM1*	746	615	865	309	172	2.4	2.0	2.8	1.8	3.6	4.3	5.0	Plasma Membrane	transmembrane receptor
8085110	*KIT*	342	248	105	66	43	5.2	3.7	1.6	1.5	5.8	8.0	2.4	Plasma Membrane	kinase
7937772	*IGF2*	381	820	632	353	91	1.1	2.3	1.8	3.9	9.0	4.2	7.0	Extracellular Space	growth factor
8062395	*NNAT*	1499	159	174	129	100	11.6	1.2	1.3	1.3	1.6	15.1	1.8	Plasma Membrane	transporter
7976012	*NRXN3*	794	83	55	43	37	18.4	1.9	1.3	1.2	2.2	21.4	1.5	Plasma Membrane	transporter
